# Distinct neuroinflammatory profiles in progressive supranuclear palsy associated with *HLA* haplotypes

**DOI:** 10.1093/braincomms/fcag084

**Published:** 2026-03-11

**Authors:** Shelley L Forrest, Sarah S Zaheer, Ain Kim, Hidetomo Tanaka, Helen Chasiotis, Jun Li, Susan H Fox, Jinguo Wang, M Carmela Tartaglia, Anthony E Lang, Gabor G Kovacs

**Affiliations:** Tanz Centre for Research in Neurodegenerative Diseases, University of Toronto, Toronto, Ontario M5T 0S8, Canada; Krembil Brain Institute, University Health Network, Toronto, Ontario M5T 0S8, Canada; Rossy Centre for PSP, Toronto Western Hospital, Toronto, Ontario M5T 2S8, Canada; Edmond J. Safra Program in Parkinson’s Disease and the Morton and Gloria Shulman Movement Disorders Clinic, Toronto Western Hospital, Toronto, Ontario M5T 2S8, Canada; Tanz Centre for Research in Neurodegenerative Diseases, University of Toronto, Toronto, Ontario M5T 0S8, Canada; Tanz Centre for Research in Neurodegenerative Diseases, University of Toronto, Toronto, Ontario M5T 0S8, Canada; Tanz Centre for Research in Neurodegenerative Diseases, University of Toronto, Toronto, Ontario M5T 0S8, Canada; Tanz Centre for Research in Neurodegenerative Diseases, University of Toronto, Toronto, Ontario M5T 0S8, Canada; Rossy Centre for PSP, Toronto Western Hospital, Toronto, Ontario M5T 2S8, Canada; Edmond J. Safra Program in Parkinson’s Disease and the Morton and Gloria Shulman Movement Disorders Clinic, Toronto Western Hospital, Toronto, Ontario M5T 2S8, Canada; Division of Neurology, University of Toronto, Toronto, Ontario M5T 1A8, Canada; Laboratory Medicine Program, University Health Network, Toronto, Ontario M5G 2C4, Canada; Department of Laboratory Medicine and Pathobiology, University of Toronto, Toronto, Ontario M5S 1A8, Canada; Tanz Centre for Research in Neurodegenerative Diseases, University of Toronto, Toronto, Ontario M5T 0S8, Canada; Krembil Brain Institute, University Health Network, Toronto, Ontario M5T 0S8, Canada; Rossy Centre for PSP, Toronto Western Hospital, Toronto, Ontario M5T 2S8, Canada; Division of Neurology, University of Toronto, Toronto, Ontario M5T 1A8, Canada; Tanz Centre for Research in Neurodegenerative Diseases, University of Toronto, Toronto, Ontario M5T 0S8, Canada; Krembil Brain Institute, University Health Network, Toronto, Ontario M5T 0S8, Canada; Rossy Centre for PSP, Toronto Western Hospital, Toronto, Ontario M5T 2S8, Canada; Edmond J. Safra Program in Parkinson’s Disease and the Morton and Gloria Shulman Movement Disorders Clinic, Toronto Western Hospital, Toronto, Ontario M5T 2S8, Canada; Division of Neurology, University of Toronto, Toronto, Ontario M5T 1A8, Canada; Tanz Centre for Research in Neurodegenerative Diseases, University of Toronto, Toronto, Ontario M5T 0S8, Canada; Krembil Brain Institute, University Health Network, Toronto, Ontario M5T 0S8, Canada; Rossy Centre for PSP, Toronto Western Hospital, Toronto, Ontario M5T 2S8, Canada; Edmond J. Safra Program in Parkinson’s Disease and the Morton and Gloria Shulman Movement Disorders Clinic, Toronto Western Hospital, Toronto, Ontario M5T 2S8, Canada; Division of Neurology, University of Toronto, Toronto, Ontario M5T 1A8, Canada; Laboratory Medicine Program, University Health Network, Toronto, Ontario M5G 2C4, Canada; Department of Laboratory Medicine and Pathobiology, University of Toronto, Toronto, Ontario M5S 1A8, Canada

**Keywords:** cytotoxic T cells, *HLA*, machine learning, microglia, progressive supranuclear palsy

## Abstract

Progressive supranuclear palsy is a neurodegenerative four-repeat tauopathy characterized by atypical parkinsonism and cognitive behavioural changes and a relatively uniform neuropathology. Building on prior identification of rare *HLA* (human leukocyte antigen) haplotypes in progressive supranuclear palsy, this study investigates whether these haplotypes correlate with distinct clinical and immunopathological phenotypes. In addition to retrospective collection of clinical data, we evaluated T and B cells, microglia and phosphorylated-tau (p-Tau) cytopathologies in 32 progressive supranuclear palsy cases. Machine learning was used to analyse whether pathological variables and their ratios, or the sequence of clinical symptoms are different *HLA*-defined groups, including one linked to narcolepsy (DRB1*15:01-DQB1*06:02). Neuropathology revealed regional differences in the severity of microglia load, density of cytotoxic T cells and p-Tau cytopathologies between groups. Machine learning revealed that specific ratios of neuroinflammatory markers reliably distinguished *HLA* haplotypes. Additionally, symptom progression sequences varied by *HLA* haplotype, suggesting a potential effect of neuroinflammatory profiles on disease trajectory. These findings support the notion that progressive supranuclear palsy pathology might be associated with various aetiological–pathogenic events rarely including targetable autoimmune mechanisms as well. The *HLA* haplotype-dependent diversity of neuroinflammatory markers should be evaluated in clinical and biomarker studies in, and beyond, progressive supranuclear palsy to understand its relevance for patient stratification in disease modifying therapy trials.

## Teaser

Progressive supranuclear palsy is a four-repeat tauopathy with atypical parkinsonism and cognitive changes. Forrest *et al*. report that rare haplotypes of human leukocyte antigen *(HLA)* associate with distinct clinical and neuropathological profiles of tau and immune cell markers. Machine learning revealed that specific ratios of neuroinflammatory markers reliably distinguished haplotypes.

## Graphical Abstract

**Graphical Abstract:**
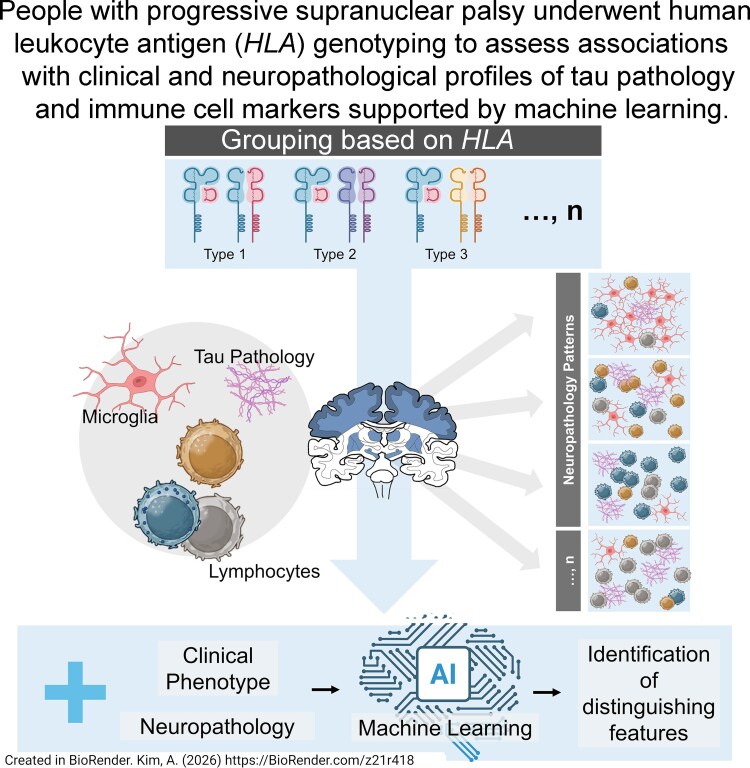

## Introduction

Progressive supranuclear palsy is a neurodegenerative four-repeat (4R) tauopathy associated with the accumulation of phosphorylated-tau (p-tau) in neurons, oligodendrocytes and astrocytes.^1^ Progressive supranuclear palsy is considered a homogeneous disorder based on hallmark neuropathological features^1^ and similar filament structure of misfolded tau reported in six progressive supranuclear palsy cases examined to date.^2^ However, clinical phenotypes associated with progressive supranuclear palsy pathology vary and include syndromes in the spectrum of atypical parkinsonism and cognitive behavioural changes.^3^ Clinical variability is associated with differences in the distribution patterns of p-tau cytopathologies^4^ and in cases with Richardson syndrome, a six-tiered neuropathological staging system was introduced.^4^

Traditionally, neurodegenerative diseases are defined by neuronal loss not explained by infection, neoplasia or toxins.^5^ However, neuroinflammation, defined as microglial and astrocytic activation and leukocyte infiltration, is increasingly recognized as a key component in disease progression and therapeutic targeting.^6^ Progressive supranuclear palsy-type pathology overlaps with some neuropathological features described in IgLON5 autoimmune encephalitis-related tauopathy^7^ and the 3R and 4R tauopathy, postencephalitic parkinsonism.^8^ Interestingly, recent studies using immunostainings for lymphocytes have shown that CD8 cytotoxic T cells are consistently present in the midbrain in progressive supranuclear palsy and are more prominent than observed in other neurogenerative parkinsonisms.^9,10^ These autopsy studies were complemented by bodily fluid studies such as cytokine profiles^11,12^ or shift in peripheral CD4- and CD8-positive T-cell populations.^12^

Despite these insights, variability in neuroinflammatory markers across progressive supranuclear palsy cases remains unexplained. Given the role of the human leukocyte antigen (*HLA*) locus in autoimmune diseases,^13^ we hypothesized that *HLA* haplotypes might be associated with distinct neuroinflammatory responses in progressive supranuclear palsy. Our recent study identified the DRB1*15:01-DQB1*06:02 haplotype and DQB1*06:01/06:02 alleles as strongly associated with progressive supranuclear palsy.^14^ In the present study, we mapped p-tau pathology and inflammatory markers in progressive supranuclear palsy brains stratified by *HLA* haplotype to explore our hypothesis.

## Material and methods

### Clinical information

Clinical data was analysed from a cohort of 32 patients clinically diagnosed with progressive supranuclear palsy, comprising 11 retrospective cases and 21 prospective cases obtained from the Rossy PSP Centre at the University Health Network (UHN). Demographic variables included sex and age at death. Disease duration was calculated from the time of onset of the first reported symptom to the time of death. We focused on symptoms explicitly documented in the clinical records; for details, see online Supplementary file Methods.

### Neuropathology and immunohistochemistry

The brain autopsy tissue was collected with informed consent according to the Declaration of Helsinki and with approval from the UHN Research Ethics Board (Protocol Nr. 20-5258). A systematic neuropathological examination was conducted following established diagnostic criteria, including assessment of vascular pathology.^15-17^ For all cases, *HLA* genotyping was available as reported.^14^ To evaluate inflammatory markers, we focused on brain regions typically affected in early to moderate stages of progressive supranuclear palsy; details on these and on antibodies and procedures used for digital pathology and morphometry analysis are provided in the online Supplementary file Methods and Tables S1 and S2 and Figs. S1–S3.

### Statistical analysis

Chi^2^ test was used to compare the frequency of presenting clinical symptoms and the level of vascular pathology using the vascular cognitive impairment neuropathology guidelines (VCING).^17^ Kruskal–Wallis and Mann–Whitney U tests were performed to compare total p-tau load; microglia load and CD8, CD4, CD3 and CD20 cell densities; semiquantitative p-tau cytopathology scores; grades of small vessel pathology; ratios of neuropathological variables; duration of illness; and age at death between groups and between regions in the same groups. The Spearman correlation test was used to compare the association between pathological variables, duration of illness and age at death. To correct for the effect of progressive supranuclear palsy neuropathological stage,^4^ Alzheimer’s disease neuropathologic change (ADNC) level,^18^ Lewy body disease (LDB) stage,^19^ duration of illness and age at death on the differences between groups, we used the non-parametric analysis of covariance (ANCOVA) and linear regression for cases with progressive supranuclear palsy stage ≥3.

### Machine learning analysis

Four separate datasets were analysed: quantification of inflammatory marker-based pathology, cytopathology, sequence of clinical symptoms and the ratios of the inflammatory marker-based pathology. For all datasets except the sequence of clinical symptoms, permutation importance and treeSHapley Additive exPlanations (SHAP) were analysed following random forest classifier with train-test split on normalized, raw data; for details see online Supplementary file Methods.

## Supplementary Material

fcag084_Supplementary_Data

## Figures and Tables

**Figure 1:**
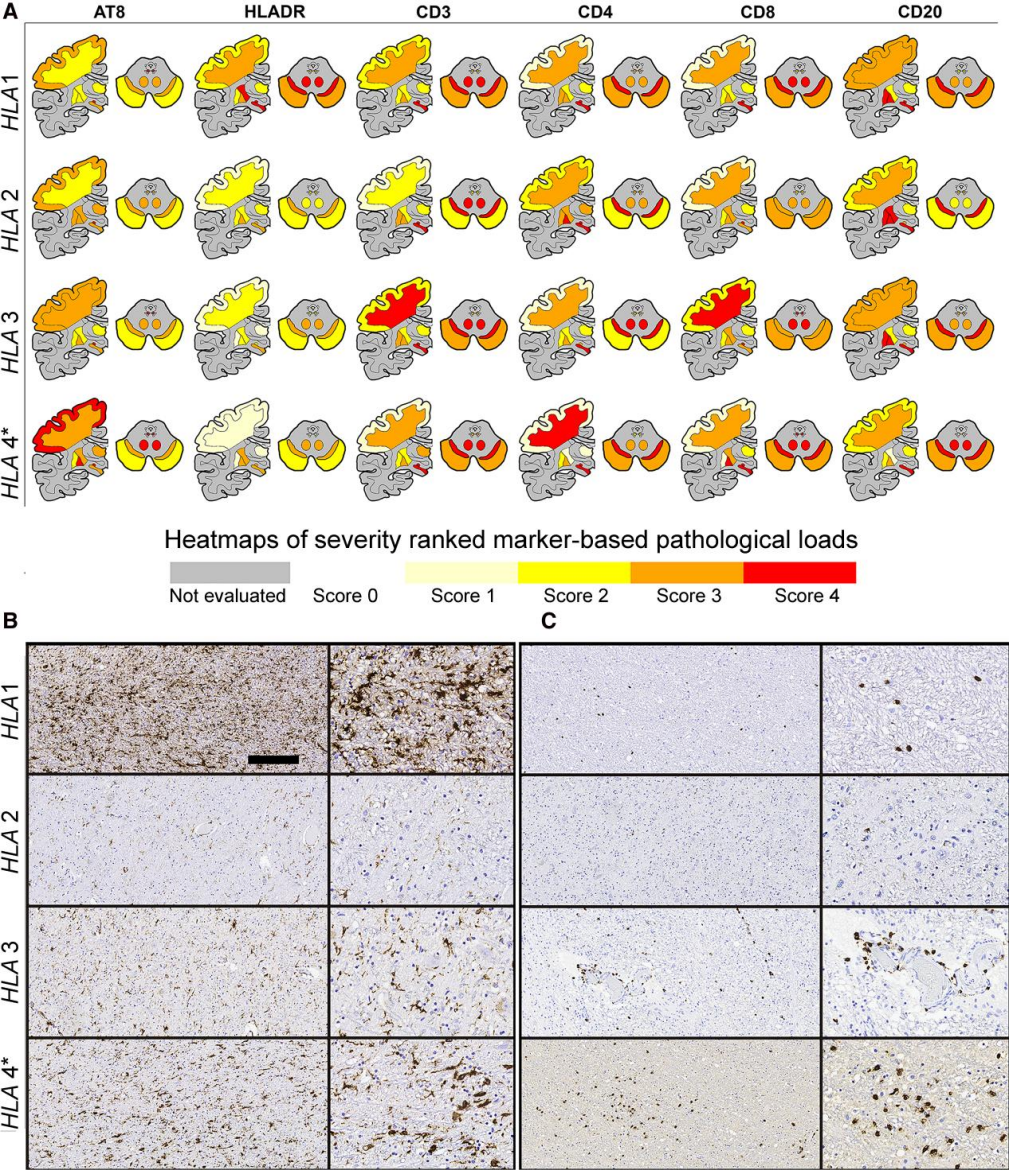
**Heatmaps of severity ranked marker-based pathological loads in *HLA* haplotypes.** (**A**) Quantification of marker-based immunoreactivity in the globus pallidus, internal capsule, putamen, motor cortex and underlying white matter, medial nucleus, subthalamic nucleus, peduncle, substantia nigra, superior cerebellar peduncle and oculomotor complex was converted to scores. The percentages of digital quantifications were standardized using z-score normalization, averaged by region for each marker in each *HLA* haplotype (*n* = 12 for *HLA* 1, *n* = 11 for *HLA* 2 and N = 8 for *HLA* 3; *HLA* 4* represents values from a single case only, DRB1*10:01-DQB1*05:01) and then divided into quartile ranks where 0 = no immunoreactivity, 1 = low, 2 = intermediate, 3 = high and 4 = very high. Regions on the heatmap template were marked using a colour scale bar (score 0 = white, score 1 = light yellow, score 2 = yellow, score 3 = orange, score 4 = red). Regions not evaluated are indicated in grey. (**B**) Representative images of the microglia staining (HLA-DR) and (**C**) CD8 immunostaining in the red nucleus/superior cerebellar peduncle area with lower (left side of **B** and **C**) and higher magnification (right side of **B** and **C**). Bar in upper image of **B** indicates 150 μm for the low and 80 μm for the high magnification images.

**Figure 2:**
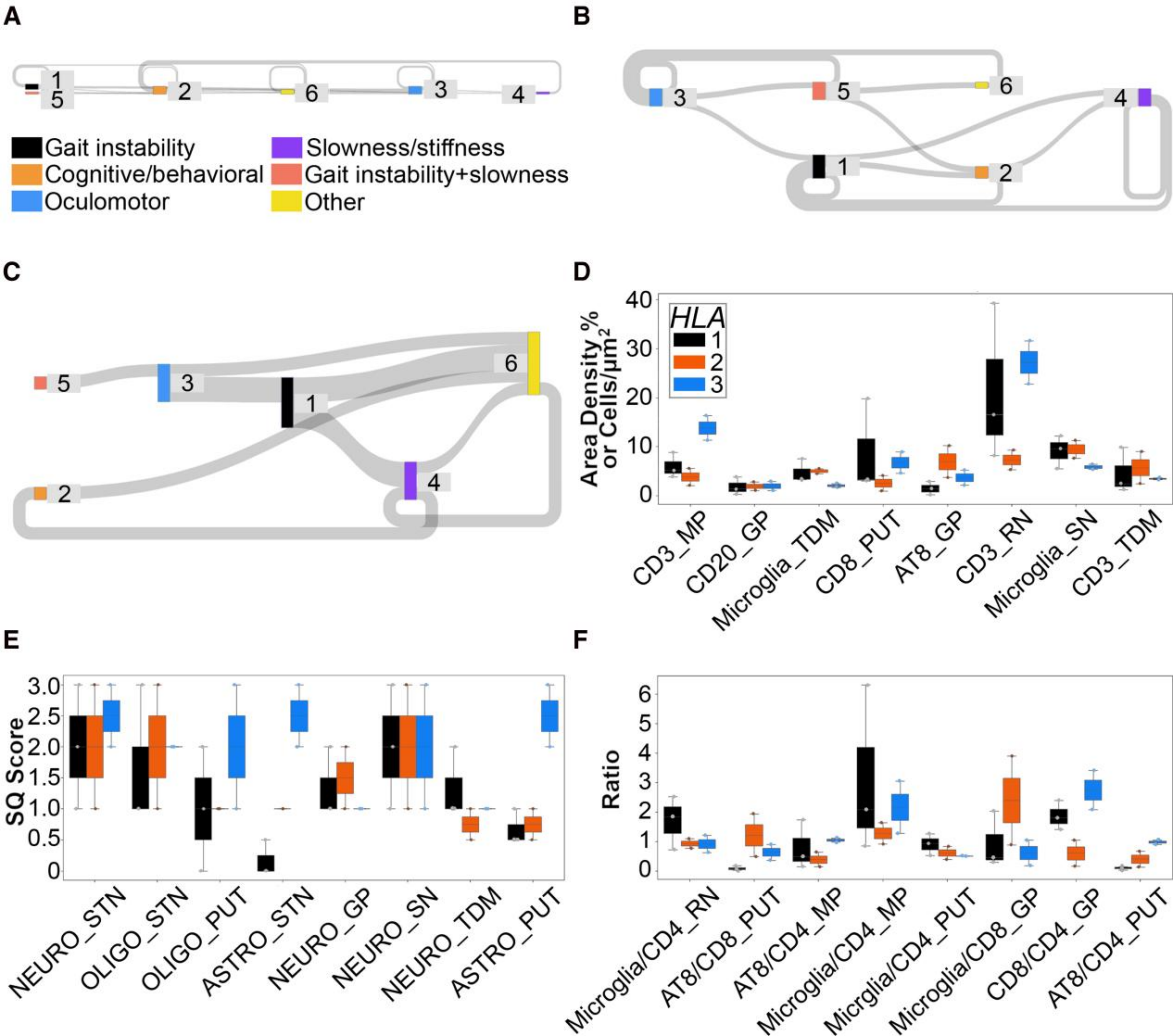
**Visualization of different clinical symptom progressions and top features that distinguish *HLA* haplotypes.** Transition matrices visualized with Sankey identified: (**A**) less diverse progression of symptoms in *HLA* group 1, (**B**) more diverse transitions in *HLA* group 2 and (**C**) symptoms most commonly leading to further branches in *HLA* group 3. Colour coding of clinical symptoms in panel A applies to B and C. Permutation importances and treeSHAP were used to identify differences in distinct features between groups using: (**D**) p-tau total load- and inflammatory marker-based pathological features, (**E**) p-tau cytopathology and (**F**) ratio of p-tau total load- and inflammatory marker-based pathology in distinct regions. Panels D–F are for descriptive visualization only and represent different features that were selected as the overlapping highest-ranking features identified by both permutation importance and treeSHAP from the held-out test-set (*n* = 7), emphasizing the most discriminative pathological features across *HLA* groups. Boxplots show the median, interquartile range and whiskers that represent the observed range of the data excluding the outliers. Individual point represents one case and appears once per feature (Online Supplementary Methods). Abbreviations: ASTRO, astroglial-tau; GP, globus pallidus; MP, midbrain peduncles; NEURO, neuronal-tau; OLIGO, oligodendroglial-tau; PUT, putamen; RN, red nucleus; SN, substantia nigra; SQ, semiquantitative; STN, subthalamic nucleus; TDM, thalamic dorsomedial nucleus.

**Figure 3:**
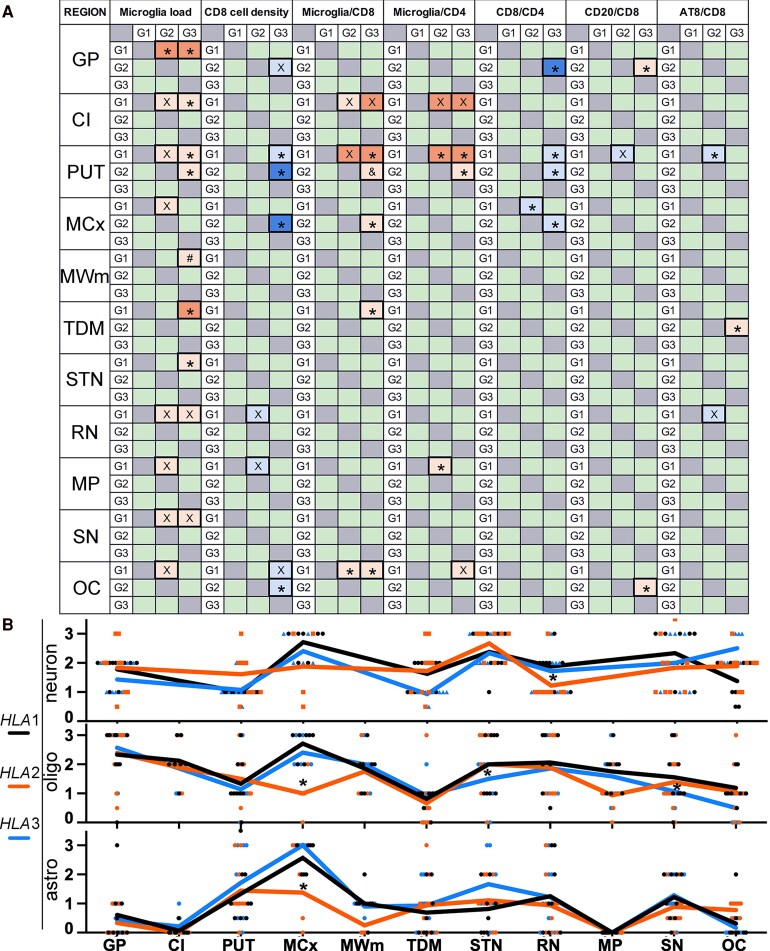
**Summary of non-parametric analysis of covariance (ANCOVA) test results.** (**A**) Comparison of haplotype groups (G1, 2, 3) in anatomical regions following ANCOVA test. Orange colour indicates that the group in the horizontal row shows a higher value for the representative variable (dark orange colour indicates *P* < 0.01; light orange colour indicates *P* < 0.05). Blue colour indicates that the group in the vertical columns shows a higher value for the representative variable (dark blue colour indicates *P* < 0.01; light blue colour indicates *P* < 0.05). X indicates significance only when corrected for age at death, progressive supranuclear palsy stage, ADNC but not duration of illness and not for stage of Lewy body pathology; * indicates significance when corrected for age, progressive supranuclear palsy, ADNC, duration of illness and for stage of Lewy body pathology; & indicates significance following correction for age, duration, ADNC, progressive supranuclear palsy and Lewy body pathology stage but not for correction for age, progressive supranuclear palsy, ADNC only; # indicates significance following correction for age, duration, ADNC, progressive supranuclear palsy but not after correction for Lewy body pathology stage. Microglia and AT8 represents area density; CD8, CD4 and CD20 represent cell density/square micrometre. The number of cases (N) varied across brain regions and *HLA* groups, with group 1 showing N = 9 for most regions except MCx, MWm, STN and OC (N = 8); group 2 showing N = 9 for most regions except CI, STN and OC (N = 8); and group 3 showing N = 7 for MP, SN, RN and OC, N = 6 for GP, PUT, TDM, CI and STN and N = 5 for MCx and MWm. *HLA group 4 represents values from a single case only (DRB1*10:01-DQB1*05:01). (**B**) Graphs of mean values of scatterplots of p-tau cytopathology scores in different brain regions demonstrated for distinct *HLA* groups (see text for details). Graphs show the p-tau-immunopositive neuronal, oligodendroglia and astroglia cytopathology scores (0–3.5) across brain regions in cases with progressive supranuclear palsy stage ≥3. *HLA* groups are shown in different colours and show different patterns. *HLA* groups are shown in different colours and show different patterns: *HLA* group 1 = black (*n* = 9), group 2 = orange (*n* = 9) and group 3 = blue (*n* = 7). Following correction for age at death, duration of illness, progressive supranuclear palsy stage and ADNC, based on ANCOVA test significant values (*P* < 0.05) are indicated with an * (oligodendrocytic: groups 1 > 2 and 3 > 2, *P* = 0.043 and 0.024, respectively, and astrocytic: group 3 > 2, *P* = 0.020) in the motor cortex; oligodendrocytic: group 2 > 3, *P* = 0.030 and group 2 > 3, *P* = 0.035 in the subthalamic nucleus and substantia nigra, respectively; and neuronal: group 1 > 2 and 3 > 2, *P* = 0.022 and 0.033, respectively, in the red nucleus. Abbreviations: GP, globus pallidus; CI, capsula interna; PUT, putamen; MCX, motor cortex; MWM, motor white matter; TDM, thalamic dorsomedial nucleus; STN, subthalamic nucleus; RN, red nucleus; MP, midbrain peduncles; SN, substantia nigra; OC, oculomotor complex.

**Figure 4:**
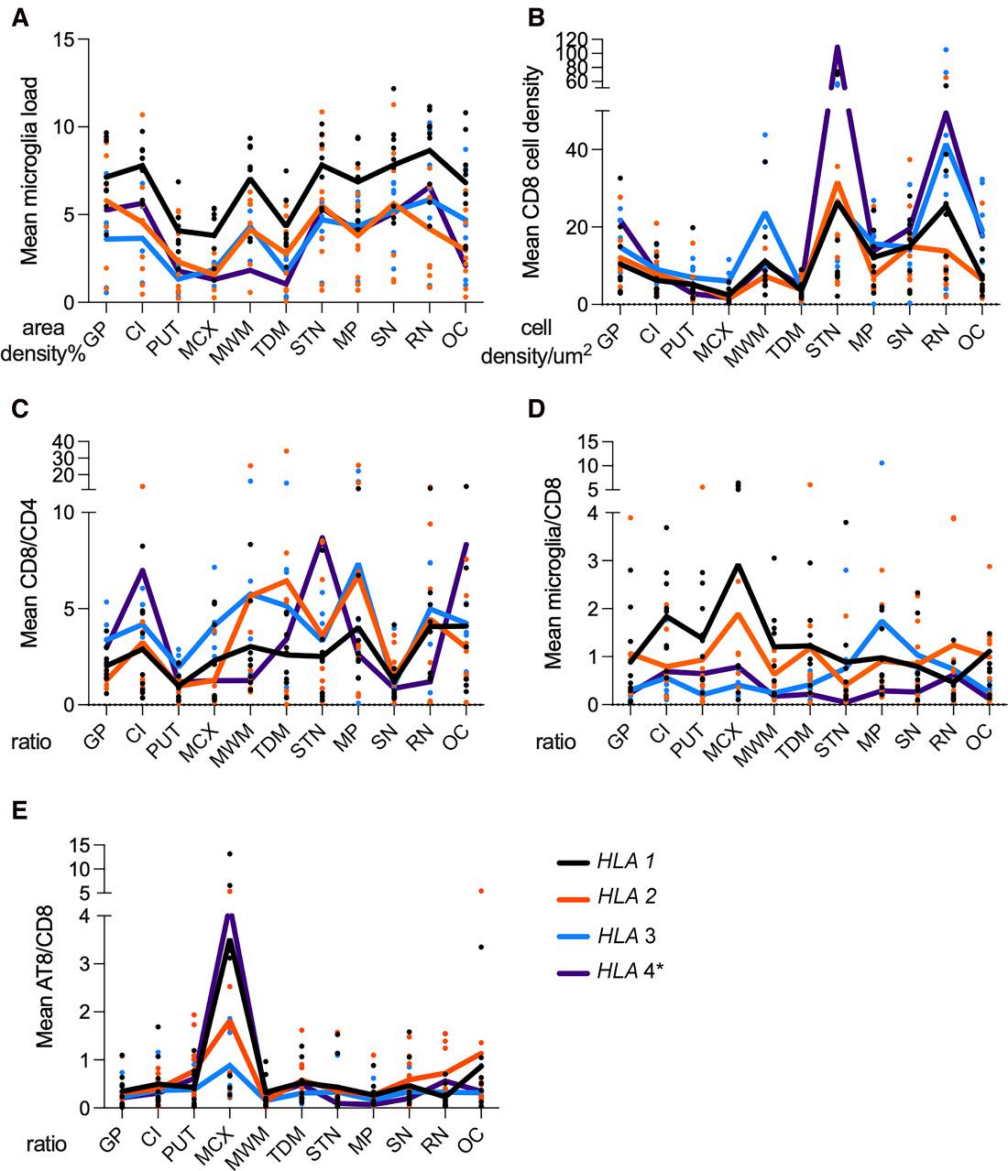
**Microglia and inflammatory cell load and their ratios across anatomical regions in the different *HLA* haplotypes.** Graphs show a descriptive lesion profile (no statistical test; for analysis of covariance, ANCOVA, test results, see Fig. 3A) the microglia (**A**) and CD8 cell (**B**) load expressed as area and cell density, respectively, and the ratio of CD8/CD4 cells (**C**), microglia/CD8 cells (**D**) and AT8/CD8 cells (**E**) across brain regions expressed as the cell density in progressive supranuclear palsy stage ≥3 cases. *HLA* haplotype groups are shown in different colours and show different patterns across brain regions. The number of cases (N) varied across brain regions and *HLA* groups, with group 1 showing N = 9 for most regions except MCx, MWm, STN and OC (N = 8); group 2 showing N = 9 for most regions except CI, STN and OC (N = 8); and group 3 showing N = 7 for MP, SN, RN and OC, N = 6 for GP, PUT, TDM, CI and STN and N = 5 for MCx and MWm. *HLA group 4 represents values from a single case only (DRB1*10:01-DQB1*05:01). Dots represent individual case values within each group, and lines indicate group means and for the single case (*HLA* group 4) connecting regional values. Abbreviations: GP, globus pallidus; CI, capsula interna; PUT, putamen; MCX, motor cortex; MWM, motor white matter; TDM, thalamic dorsomedial nucleus; STN, subthalamic nucleus; RN, red nucleus; MP, midbrain peduncles; SN, substantia nigra; OC, oculomotor complex.

## Data Availability

Data will be shared upon reasonable request from any qualified investigator. Algorithms used in this study are publicly available in scikit-learn library (https://scikit-learn.org/) and SHAP (https://shap.readthedocs.io).

## Results

### Grouping of cases

Three groups were defined based on *HLA* haplotypes:^14^ (i) 12 cases with a haplotype associated with narcolepsy (*DRB1**15:01-*DQB1**06:02), (ii) 11 cases with other *DQ5-DQ6* haplotypes and (iii) 8 cases with various haplotypes frequent in the general population. In addition, we examined one case with the haplotype frequent in IgLON5-associated encephalitis (*DRB1**10:01-*DQB1**05:01) and a case suspected to have postencephalitic parkinsonism showing 3R and 4R tau pathology in the substantia nigra.^20^ These two cases were excluded from machine learning algorithms and comparative statistics.

### Clinical and neuropathological characteristics of *HLA* haplotypes

Age at death (76.11 + 2.36, 77.22 + 2.14 and 75.86 + 2.04 years; *P* = 0.68) and duration of illness (6.33 + 0.60, 10.10 + 2.25 and 8.00 + 1.34 years, *P* = 0.23) in cases with progressive supranuclear palsy stage ≥3 were similar between *HLA* haplotype groups 1–3, respectively; furthermore, the chi^2^ test did not show differences between sex distribution (*P* = 0.69) and between the distribution of ADNC categories (*P* = 0.141) or VCING levels (*P* = 0.455). Clinical and neuropathological information is summarized in Supplementary file Tables S3.

Most cases showed progressive supranuclear palsy stage ≥3 p-tau pathology in all groups; the chi^2^ test did not show differences between the distribution of stages (*P* = 0.60). Group 2 included a case compatible with limbic predominant 4R tauopathy (LNT)-type pathology overlapping with progressive supranuclear palsy-type pathology.^21^ Group 3 of our cohort consisted of various haplotypes. None of the co-pathologies differed significantly between groups (*P* > 0.2 for all). Heatmapping of the mean values of total p-tau load and various inflammatory markers revealed distinct patterns for the different groups as also observed in tissue sections (Fig. 1).

Group 1 cases were more likely to present with gait instability and stiffness compared to group 2, which showed other presenting symptoms, including cognitive/behavioural or oculomotor dysfunction more frequently (*P* = 0.08). Group 3 cases showed cognitive, oculomotor dysfunction or gait instability as the presenting symptom. Transition matrices suggest that different haplotypes have different transition sequences, where group 1 tends to have less diversity and many ‘looping’ (i.e. lack of progression), group 2 has more diverse types of symptom transitions and group 3 tends to lead to a common endpoint of ‘other’ symptoms, suggesting more branches of sequences within group 3 (Fig. 2A–C). The cosine similarity ranged from 0.16 to 0.47, and KL divergence ranged from 60 to 90, demonstrating that the different haplotypes do not share similar transition probabilities. Lastly, the permutation test showed that the sequence differences between haplotypes exceeded the differences within the same haplotype (observed separation = 0.11, *P* = 0.097), although not statistically significant.

### Distinct microglia load associated with different *HLA* haplotypes

Microglia load was higher in several regions in group 1 compared to those in group 2 and 3 (online Supplementary file Results). No significant correlation was found between progressive supranuclear palsy stage and microglia load when all regional values were pooled in groups 1 and 2 (rho = 0.11, *P* = 0.21; rho = 0.12, *P* = 0.19, respectively) in contrast to group 3 (rho = 0.56, *P* < 0.01). When examined for each region microglia load correlated with progressive supranuclear palsy stage only in group 3 in the oculomotor complex (ρ = 0.74, *P* = 0.035) and substantia nigra (ρ = 0.86, *P* = 0.006). Across all cases and regions (*n* = 324), microglia load correlated with p-tau load (ρ = 0.393, *P* < 0.001). ANCOVA revealed significant differences in microglia load between groups in the globus pallidus (group 1 > 2 and 3), putamen (groups 1 and 2 > 3), internal capsule, thalamic dorsomedial nucleus and subthalamic nucleus (group 1 > 3) (Fig. 3A). Anatomical lesion profiles varied by *HLA* haplotype (progressive supranuclear palsy stage ≥3), with the midbrain and subthalamic nucleus most affected, except in group 2 where the globus pallidus involvement was predominant (Fig. 4A; online supplemental file Tables S4 and S5). Machine learning identified two brain regions where microglia load was one of the most distinguishing features between *HLA* groups (Fig. 2D). The LNT case^21^ in group 2 showed higher p-tau and microglia load in several brain regions. The case with suspected postencephalitic parkinsonism^20^ showed lower microglia load and p-tau load in all regions, except the substantia nigra where p-tau load was the highest (online supplemental file Fig. S4).

### Differences in CD8 cytotoxic cell counts between *HLA* haplotypes

CD20-positive B cells and CD4-positive T cells showed similar densities between groups. However, CD3-positive T cells were significantly more abundant in the motor area white matter in group 3 compared to group 2. CD8-positive cytotoxic T-cell densities were higher in group 3 than in groups 1 and 2 across multiple regions (online Supplementary file Results). When pooling all cases (*n* = 32), densities of CD20, CD3, CD4 and CD8 cells correlated positively with p-tau load (rho = 0.114, 0.385, 0.189, 0352, respectively; *P* = 0.040 for CD20 and *P* < 0.001 for CD3, CD4 and CD8). CD8 cell density correlated positively with microglia load in the pooled total cohort and in each *HLA* group (*P* < 0.01 for all) even after correcting for age, ADNC level and progressive supranuclear palsy stage. When evaluating correlation in *HLA* groups in different brain regions revealed significance that was lost when correcting for multiple variables (*P* > 0.05) (online Supplementary file Results). ANCOVA analysis revealed significant differences only for CD8 cells, particularly in the putamen, motor cortex and oculomotor complex, with group 3 showing higher densities (Fig. 3A). Anatomical distribution varied by *HLA* haplotype in progressive supranuclear palsy stage ≥3 cases (Fig. 4B). CD8 cells were most prevalent in the subthalamic nucleus, while CD4 cells were concentrated in the substantia nigra and subthalamic nucleus. The globus pallidus had higher T-cell involvement in group 2 (online supplemental file Tables S4 and S5). Machine learning identified five brain regions where either CD3, CD8 or CD20 cell densities were one of the most distinguishing features between *HLA* groups (Fig. 2D). Notably, the LNT case mirrored typical CD4 and CD8 patterns of group 2, while the suspected postencephalitic parkinsonism case showed reduced T-cell densities (online supplemental file Fig. S4).

### p-Tau load and cytopathology differences between *HLA* haplotypes

Total p-tau load across brain regions did not differ significantly between *HLA* haplotype groups, even after adjusting for age at death, illness duration, progressive supranuclear palsy stage and ADNC (*P* > 0.2). However, regional distribution patterns of total p-tau load varied between cases with progressive supranuclear palsy stage ≥3 (online supplemental file Tables S4 and S5).

Mean neuronal, oligodendrocytic and astrocytic p-tau cytopathology scores showed variability between brain regions. The ANCOVA test confirmed significant differences of p-tau cytopathology scores for oligodendrocytic (groups 1 > 2 and 3 > 2, *P* = 0.043 and 0.024, respectively) and astrocytic (group 3 > 2, *P* = 0.020) in the motor cortex, oligodendrocytic in the subthalamic nucleus (group 2 > 3, *P* = 0.030) and substantia nigra (group 2 > 3, *P* = 0.035) and neuronal in the red nucleus (group 1 > 2 and 3 > 2, *P* = 0.022 and 0.033, respectively) (Fig. 3B). Machine learning identified one brain region where total p-tau load was one of the most distinguishing features between *HLA* groups (Fig. 2D). In terms of cytopathologies, all cell types showed distinguishing patterns across brain regions (Fig. 2E). Regional distribution patterns of total p-tau load varied between cases with progressive supranuclear palsy stage ≥3 (online supplemental file Tables S4 and S5).

### Distinct ratios of pathology variables between *HLA* haplotypes

Ratios of neuropathological markers varied significantly across brain regions (online Supplementary file Results). The ANCOVA test revealed notable differences (Fig. 3A). Microglia/CD8 ratios were lower in group 3 compared to groups 1 and 2 in the putamen, motor cortex, thalamic dorsomedial nucleus and oculomotor complex. Microglia/CD4 ratios were highest in group 1 across the putamen and midbrain peduncle. CD8/CD4 ratios were elevated in group 3 in the globus pallidus, putamen and motor cortex. CD20/CD8 ratios were higher in group 2 in the globus pallidus and oculomotor complex. AT8/CD8 ratios were increased in group 2 in the putamen and thalamic dorsomedial nucleus.

Distinct anatomical patterns emerged across *HLA* haplotypes (progressive supranuclear palsy stage ≥3) (Fig. 4C–E), with greater variability in ratios than in single markers (online supplemental file Tables S6 and S7). Machine learning identified various ratios of neuropathology variables in four brain regions as distinguishing features between *HLA* groups (Fig. 2F). The LNT case showed similar regional distribution of ratios as group 2 except for microglia/CD8, microglia/CD4 and CD8/CD4 (online supplemental file Fig. S5). The case with suspected postencephalitic parkinsonism showed similar patterns as group 3 except for the CD20/CD8 value, which deviated in some regions (online supplemental file Fig. S5).

## Discussion

This study introduces a novel framework for interpreting neuroinflammatory responses in progressive supranuclear palsy, with broader implications for other neurodegenerative diseases. It demonstrates that the distribution and interaction of inflammatory cells are strongly associated with an individual's *HLA* haplotype. Analogously to the finding of the same tau filament fold in chronic traumatic encephalopathy and other conditions interpreted as environmentally induced or of genetic cause,^22^ these results indicate that common neuropathology and tau filament fold can be induced by various aetiologies. The possibility that tau derived peptides, rather than misfolded tau filaments, might be presented by HLA molecules to T cells suggests a potential autoimmune mechanism in a subset of progressive supranuclear palsy cases.^14^ Though direct evidence remains limited, this was supported by finding nigral CD8 cytotoxic T cell–neuron contacts in some cases^10^ and here by showing different patterns of the neuroinflammatory response.^6^ Interestingly, progressive supranuclear palsy-type neuropathology has previously been documented in association with Iglon5 autoimmune disease,^23^ and in one case in our study, which carried the *HLA* haplotype commonly linked to Iglon5 disease, we identified distinct immune cell distribution patterns.

In this cohort, individuals with the narcolepsy-associated haplotype (DRB1*15:01-DQB1*06:02) showed increased microglial activation, while CD8 T-cell responses varied across other haplotypes, including one linked to IgLON5-associated brain disease (*DRB1**10:01-*DQB1**05:01).^7,24^ Genome-wide association studies have identified risk loci near the *HLA* region, reinforcing its relevance in progressive supranuclear palsy.^25^ Notably, the LNT case exhibited elevated microglial activity as those in group 2, while a suspected postencephalitic parkinsonism case showed reduced inflammation, except for increased CD20 B cells in certain regions, suggesting that rare subtypes may have unique inflammatory profiles beyond *HLA* influence.

Previous research has explored adaptive immunity in progressive supranuclear palsy, particularly in the frontal cortex of patients with frontotemporal dementia.^26^ Peripheral immune markers also show variability among progressive supranuclear palsy patients, aligning with the current findings.^11,12^ Importantly, differences in inflammatory pathology remained significant even after adjusting for co-existing neurodegenerative conditions. Experimental models further highlight the role of T cells in tauopathy progression,^27^ suggesting they may be more viable therapeutic targets than tau tangles themselves.

While clinical symptoms alone may not predict *HLA* haplotype, symptom progression patterns and inflammatory marker profiles offer partial insights. Certain ratios of immune markers can distinguish *HLA* haplotypes, pointing to biologically meaningful *HLA*-specific neuroinflammatory phenotypes. A limitation of this study is the reliance on retrospective clinical data and the absence of standardized clinical disease severity scores. Furthermore, *HLA* haplotypes may only partially account for the variations in clinical symptoms and pathological findings. Differences in the levels of microglia or CD8 T-cell loads may reflect external factors such as treatment or non-*HLA* genetics. Moreover, the extensive scope of the study, requiring stainings on 768 sections and 1954 subregion annotations (see online supplemental file), limited the number of cases included. Nonetheless, our findings draw attention to the importance of incorporating *HLA* haplotyping into the interpretation of neuroinflammatory responses. This approach could be extended to Parkinson’s disease and Alzheimer’s disease, where *HLA* has been studied but not linked to inflammatory markers.^28-30^

In summary, our findings advocate for patient stratification in therapeutic trials and highlight early immune modulation as a potential complementary treatment avenue in a rare subset of patients. Future genome-wide and biomarker studies should consider *HLA*-based stratification to better understand disease mechanisms and improve therapeutic targeting.
