# Supplementary material for: Distinct neuroinflammatory profiles in progressive supranuclear palsy associated with HLA haplotypes
Source: Brain Commun. 2026 Mar 11;8(2):fcag084. doi: 10.1093/braincomms/fcag084 (PMC13012215; doi:10.1093/braincomms/fcag084)
Supplement: fcag084_Supplementary_Data [file fcag084_supplementary_data.pdf]

SUPPLEMENTARY METHODS

Clinical information

Symptom chronology was assessed by identifying the first, second, and third reported symptoms in the order of their appearance. The presence of each symptom was annotated as "+" if reported, "-" if the symptom was not reported, tested and found absent, and "N/A" if the data were unavailable. To further characterize disease severity and clinical heterogeneity, we recorded additional features commonly associated with PSP, including vertical supranuclear gaze palsy, bulbar dysfunction, gait dysfunction and presence of falls, parkinsonian features, psychiatric manifestations, cognitive dysfunction and behavioral disturbances, language abnormalities, and sleep disturbances. Cases with both natural death and death via Medical Assistance in Dying (MAiD) were included.

Neuropathology

Regions examined included the midbrain (oculomotor complex, substantia nigra, cerebral peduncle, red nucleus, and superior cerebellar peduncle), posterior basal ganglia (globus pallidus, putamen, internal capsule), thalamic region (subthalamic nucleus and medial thalamic nucleus), and motor cortex (gray and white matter assessed separately). For the semiquantitative evaluation of p-tau cytopathologies we used a six-tiered approach as follows: none (0), single (0.5), mild (1), moderate (2), severe (3), extremely severe (3.5). Although total p-tau load could be quantified using software analysis, current morphometric and machine learning tools lack the specificity to reliably differentiate between astrocytic, oligodendrocytic, and neuronal p-tau pathologies, necessitating the use of a classical semiquantitative approach for the different cytopathologies. Quantification of marker-based immunoreactivity in the globus pallidus, internal capsule, putamen, motor cortex and underlying white matter, medial nucleus, subthalamic nucleus, peduncle, substantia nigra, superior cerebellar peduncle and oculomotor complex were converted to scores. The percentages of digital quantifications were standardized using z-score normalization, averaged by region for each marker in each HLA haplotype and then divided into quartile ranks where 0 = no immunoreactivity, 1 = low, 2 = intermediate, 3 = high, and 4 = very high. Regions on the heatmap template were marked using a color scale bar (score 0 = white, score 1 = light yellow, score 2 = yellow, score 3 = orange, score 4 = red). Regions not evaluated are indicated in grey. These scores were visualized using heatmaps, with a color scale ranging from white (0) to red (4). Regions not evaluated were marked in grey.

Immunohistochemistry was performed using the following primary antibodies: anti-phospho-tau (clone AT8, pSer202/Thr205, 1:1000, Thermo Fisher Scientific), microglia marker anti-HLA DR + DP + DQ (clone CR3/43, 1:200, Abcam), anti-CD3 (1:200, polyclonal, Dako/Agilent), anti-CD4 (clone EPR6855, 1:500, Abcam), anti-CD8 (clone C8/144B, 1:200, Dako/Agilent), and anti-CD20 (clone L26, 1:500, Dako/Agilent). In each case 6 immunostainings were performed in 4 paraffin embedded tissue blocks (in sum 768 sections). Immunostaining was performed using the Dako Autostainer Link 48 and EnVision FLEX+ Visualization System, according to the manufacturer's instructions.

Digital pathology and morphometry

Stained sections were digitized using the Huron TissueScope LE120 slide scanner, and images were imported into HALO software (Indica Labs) for manual annotation of subregions of interest (11 anatomical subregions) and automated quantification of AT8, microglia, CD3, CD4, CD8 and CD20 area cell density within the annotated subregions of interest. Since a few subregions were

not identifiable, in summary this resulted in 1954 annotated subregions. Neuromelanin was manually excluded from analysis in the substantia nigra. Scanned images of immunostained sections were imported into HALO software (version 3.6.4134, Indica Labs), and using the Pen Annotation tool, 11 subregions of interest were manually annotated: putamen, globus pallidus and capsula interna of the basal ganglia, gray matter and white matter of the motor cortex, medial nucleus and subthalamic nucleus of the thalamus, cerebral peduncle, substantia nigra, ruber and oculomotor complex of the midbrain (Fig. S1A). The Object Colocalization module (version 2.1.5) within HALO software was then used for the automated quantification of DAB-positive staining to determine the area densities of AT8 (Fig. S1B, C) and HLA-DR (Fig. S1D, E). Area density for AT8 and HLA-DR was expressed as the percentage of the DAB-positive area within the area of the subregion of interest. For automated counts of CD3, CD4, CD8 and CD20 cells in HALO, the detection parameters within the Object Colocalization module were initially adjusted such that only single immuno-positive cells were detected (Fig. S2A-B). Using these preliminary parameters, 14-15 randomly selected immunostained sections were analyzed and the average area of single CD3, CD4, CD8 and CD20 cells was calculated. The average area ($\mu m^2 \pm$SEM, n=number of cells counted) calculated for each immuno-positive cell was as follows: CD3 (20.96±0.05, n=63,097), CD4 (20.70±0.08, n=14,439), CD8 (26.21±0.08, n=32,980), CD20 (19.94±0.12, n=8,265). Finally, the parameters were re-adjusted such that the total area of CD3, CD4, CD8 and CD20 immuno-positivity was detected within the subregions of interest (Fig. S2C), and then this total immuno-positive area was divided by the calculated average area of a single cell to give the total number of immuno-positive cells detected within the subregion. CD3, CD4, CD8 and CD20 area density is expressed as the number of DAB-positive cells per μm^2 of the subregion of interest. Detailed parameters utilized for the detection of immuno-positive area with the Object Colocalization module can be found in Tables S1 and S2. False immuno-positive detection of neuromelanin in the substantia nigra subregion was excluded in all analyses by manually screening and circling neuromelanin in the substantia nigra using the Exclusion Annotation tool in Halo (Fig. S3).

Figure S1: Annotation of brain subregions of interest and automated detection of AT8 (p-tau) and HLA-DR (microglia marker) area density using HALO software. (A) Representative annotation of the red nucleus (purple outline), substantia nigra (yellow outline) and cerebral peduncle (green outline) in the midbrain using HALO. (B) Sample AT8 immunostaining and (C) AT8 immuno-positive area detection (green) in HALO. (D) Sample HLA-DR immunostaining and (E) HLA-DR immuno-positive area detection (green) in HALO. Scale bar = 1mm in (A), 20µm in (B-E).

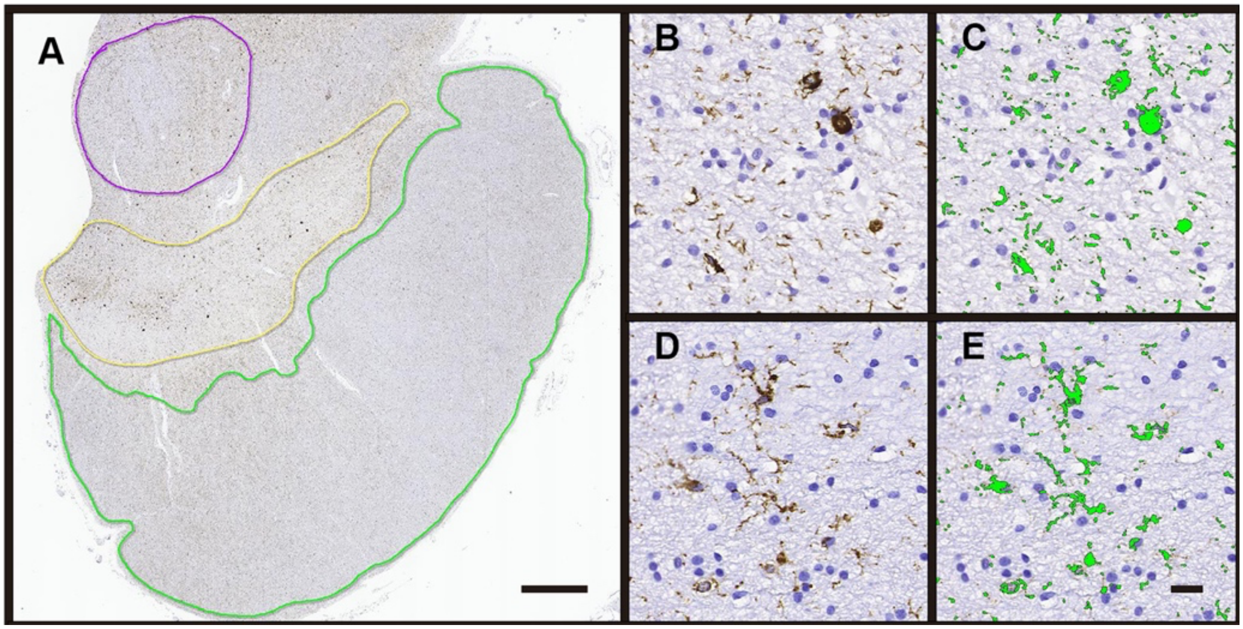

Figure S2: Automated detection of CD3 (T cell marker), CD4 (helper T cell marker), CD8 (cytotoxic T cell marker) and CD20 (B cell marker) immunoreactivity and area density calculation using HALO software. (A) Representative image of CD8 immunostaining in the capsula interna of the basal ganglia. (B) Detection parameters within the Object Colocalization module of HALO were initially adjusted such that only single immuno-positive cells were detected (green). This allowed the average area of a single immuno-positive (e.g. CD8) cell to be calculated. (C) Detection parameters were then re-adjusted so that the total immunoreactive area was detected (green). The total immunoreactive area was then divided by the average area of a single cell (calculated from B) to determine the total number of immuno-positive cells detected within the subregion of interest. Scale bar = 20µm.

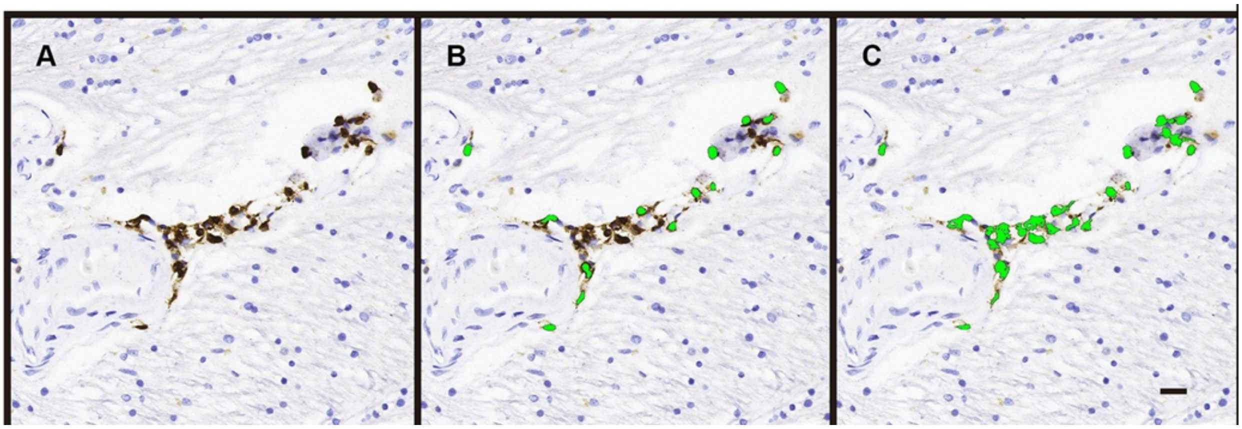

Figure S3: Method of neuromelanin exclusion from analysis in the substantia nigra by HALO software. A) Representative image of neuromelanin (black arrows) and AT8 (p-tau) immunostaining in the substantia nigra. (B) Regions of neuromelanin were manually circled (yellow/black dashed circles) and excluded from analysis using the Exclusion Annotation tool in HALO. C) AT8 immunostaining is detected (green) and neuromelanin has been excluded from analysis. Scale bar = 20µm.

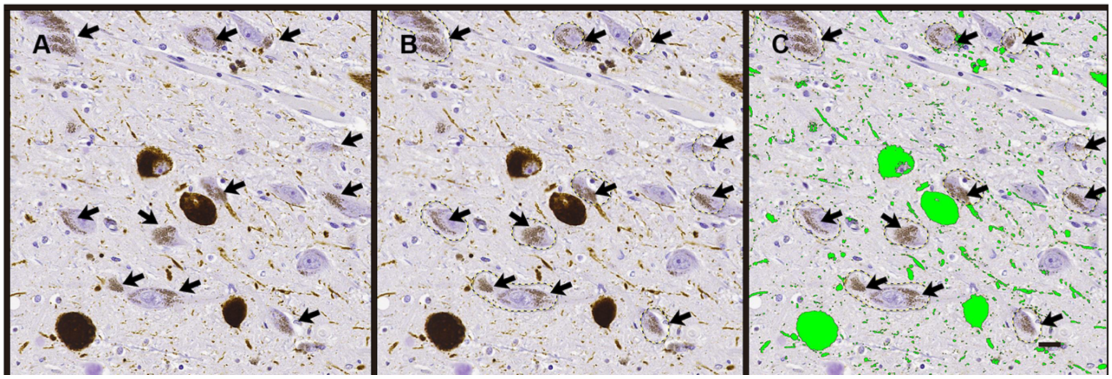

Table S1: Object Colocalization module parameters in HALO software for the automated detection of the total area of AT8, HLA-DR, CD3, CD4, CD8 and CD20 immunostaining.

Parameters	AT8	HLA-DR	CD3	CD4	CD8	CD20
**Stain Color**	0.467, 0.600, 0.773	0.467, 0.600, 0.773	0.394, 0.467, 0.509	0.394, 0.467, 0.509	0.394, 0.467, 0.509	0.394, 0.467, 0.509
**Blur Radius**	0	0	0	0	0	0
**Contrast Radius**	278.444	500	500	500	500	500
**Contrast Threshold**	0.821	0.828	0.841	0.841	0.841	0.841
**Optical Density**	0	0	0.69	0.69	0.69	0.69
**Object Size**	10, 100000	10, 100000	15, 100000	15, 100000	15, 100000	15, 100000
**Connect Length**	0	0	0	0	0	0
**Fill Holes**	False	False	True	True	True	True

Table S2: Object Colocalization module parameters in HALO software for the automated detection of area in single CD3, CD4, CD8 and CD20 immuno-positive cells.

Parameters	CD3	CD4	CD8	CD20
**Stain Color**	0.394, 0.467, 0.509	0.394, 0.467, 0.509	0.394, 0.467, 0.509	0.394, 0.467, 0.509
**Blur Radius**	11.73	22.26	18.27	15.09
**Contrast Radius**	10	9	9	9
**Contrast Threshold**	0.868	0.861	0.861	0.861
**Optical Density**	0.47	0.47	0.47	0.47
**Object Size**	10, 100000	10, 100000	10, 100000	10, 100000

**Connect Length**	0	0	0	0
**Fill Holes**	True	True	True	True

Machine learning analysis

Four separate datasets were analyzed: quantification of inflammatory marker-based pathology, cytopathology, sequence of clinical symptoms and the ratios of the inflammatory marker-based pathology. For all datasets except the sequence of clinical symptoms, permutation importance and treeSHapley Additive exPlanations (SHAP) was analyzed following RFC on normalized raw data that incorporated confounding effects including age, disease duration, PSP stage, and ADNC. Group-wise differences in the top 8 biological features were visualized across HLA haplotypes using the test split of the data (n=7). Each point represents an individual subject in the test-set, resulting in a total of 7 datapoints per feature across the different haplotypes. For the progression of clinical symptoms, where symptoms were categorized (0=none, 1=gait instability including falls, 2=cognitive/behavioral, 3=oculomotor, 4=slowness or stiffness, 5=early combination of gait instability and slowness and 6=other), transition matrices were generated and visualized using Sankey with significance reported using permutation. Algorithms used in this study are publicly available in scikit-learn library (https://scikit-learn.org/) and SHAP (https://shap.readthedocs.io). All visualizations were created using the matplotlib library (https://matplotlib.org) and plotly (https://pypi.org/project/plotly/).

SUPPLEMENTARY RESULTS

Table S3. Clinical and neuropathological data of the cohort examined grouped based on HLA haplotypes.

HLA-Haplotype	Sex	Clinical diagnosis	Age at Death	Duration (years)	1st Sy	2nd Sy	3rd Sy	PSP stage	AGD stage	ADNC	LBD type	LBD stage	LATE	CAA	VCING	FVS	BGVS
DRB1*15:01-DQB1*06:02	F	PSP-CBS	73	3	SW	C	S	4	1	L	-	-	-	-	L	2	1
	M	TES	73	6	G	C	-	5	3	P	-	-	-	-	L	2	2
	M	PSP-RS	76	5	G	O	-	3	1	P	-	-	-	-	L	0	1
	F	PSP-RS	83	9	G	O	S	2	-	P	-	-	-	-	L	2	3
	M	PSP-Pa	87	N/A	G	S	SW	2	-	P	B	1	-	-	L	1	1
	M	PSP-RS	68	4	G	N/A	-	5	-	P	-	-	-	-	L	2	2
	M	PSP-RS	73	4	G+S	C	D	4	2	I	-	-	-	+	L	2	2
	F	PSP-RS	74	8	G+S	O	C	4	1	I	-	-	-	-	L	1	2
	M	CBS	93	4	PPA	CBS	-	4	2	I	-	-	1	-	L	2	3
	M	PPA and RS	77	8	PPA	O	-	4	1	L	-	-	-	-	L	2	2
	F	PDD + PSP	90	22	S	C	G	1	-	I	Li	4	-	+	L	2	2
	M	PSP-Pa	79	7	S	C	O	4	-	L	B	1	-	+	I	3	2
Other DQ5/6 haplotypes	F	CD+Falls	78	4	C	G	O	3	-	H	-	-	-	+	I	3	3
	F	Pa+CD	77	18.5	C	S	N/A	3	1	L	B	2	1	-	H	3	3
	M	PSP-RS	72	8	G+S	O+C	N/A	5	-	P	B	2	-	-	L	2	2
	F	PSP-RS	69	5	G+S	SW	O	2	3	P	-	-	-	-	L	1	1
	M	Pa+CD	79	N/A	N/A	N/A	N/A	3	-	L	-	-	-	+	L	3	2
	F	PSP-RS	79	N/A	N/A	N/A	N/A	5	-	L	B	3	-	+	I	2	2
	M	CD	69	N/A	C	N/A	N/A	4	-	L	B	2	-	+	L	2	2
	M	PSP-RS	84	11	O	G+S	C	5#	-	L	-	-	-	+	I	2	3
	M	PSP-RS	69	6	O	G	S	3	3	L	-	-	-	-	L	3	3
	M	Pa+CD	88	N/A	C	G+S	O	4	-	H	-	-	3	-	L	1	2
	F	Pa	73	11	S	G	C	2	1	P	-	-	-	-	L	3	3
Other haplotypes	M	Pa+CD	70	29	C	O	S	##	-	L	A	-	-	-	L	2	1
	M	Pa+CD	81	5	C	G+O	S	3	-	I	B	3	-	+	L	2	3
	F	PSP-RS	70	9	G	S	C	5	-	P	-	-	-	-	L	2	2
	M	PSP-RS	72	9	G	S	C	4	-	L	-	-	-	-	L	2	3
	M	PSP-RS	72	11	G+S	O+C	SW	4	1	P	-	-	-	-	L	3	3
	F	LM	84	N/A	N/A	N/A	N/A	3	-	L	-	-	-	+	I	2	2
	M	PSP-RS	73	3	O	G	S	4	3	P	-	-	-	+	I	1	2
	M	PSP-RS	79	9	O	G	SW	5	-	P	-	-	-	-	L	1	2
DRB1*10:01-DQB1*05:01	M	PSP-RS	71	6	C	G	S	3	-	P	-	-	-	-	L	2	2

Abbreviations: A: amygdala, ADNC: Alzheimer's disease neuropathologic change, AGD: argyrophilic grain disease, B: brainstem, BGVS: basal ganglia vessel score, CAA: cerebral amyloid angiopathy, CBS: corticobasal syndrome, C: cognitive, CD: cognitive decline, D: dystonia, F: female, FVS: frontal vessel score, G: gait, H: high, I: intermediate, L: low, Li: limbic, LATE: limbic age-related TDP-43 encephalopathy, LBD: Lewy body disease, LM: limited mobility, M: male, N/A: not available, O: oculomotor, PSP: progressive supranuclear palsy, P: primary age-related tauopathy, Pa: parkinsonism, PDD: Parkinson's disease dementia, PPA: primary progressive aphasia, RS: Richardson syndrome, S: slowness/stiffness, SW: swallowing difficulty/Slurred speech, TES: traumatic encephalopathy syndrome. +: present, -: not present. #Indicates a case with limbic predominant 4R tauopathy type pathology overlapping with PSP type pathology.[1] ##: case reported to be compatible with postencephalitic parkinsonism[2]. PSP stage according to Kovacs et al.,[3] AGD stage according to Saito et al.,[4] ADNC according to Montine et al.,[5] LBD type according to Attems et al.,[6] LBD stage according to Braak et al.,[7] LATE according to Nelson et al,[8] vascular cognitive impairment neuropathology guidelines (VCING) according to Skrobot et al.[9]

References:

1. Forrest SL, Tartaglia MC, Kim A*, et al*. Progressive Supranuclear Palsy Syndrome Associated With a Novel Tauopathy: Case Study. *Neurology*. Dec 12 2022;99(24):1094-1098. doi:10.1212/WNL.0000000000201485
2. Tanaka H, Lee S, Martinez-Valbuena I*, et al*. Ageing-related tau astrogliopathy severely affecting the substantia nigra. *Neuropathol Appl Neurobiol*. Aug 2024;50(4):e13000. doi:10.1111/nan.13000
3. Kovacs GG, Lukic MJ, Irwin DJ*, et al*. Distribution patterns of tau pathology in progressive supranuclear palsy. *Acta Neuropathol*. Aug 2020;140(2):99-119. doi:10.1007/s00401-020-02158-2
4. Saito Y, Ruberu NN, Sawabe M*, et al*. Staging of argyrophilic grains: an age-associated tauopathy. *J Neuropathol Exp Neurol*. Sep 2004;63(9):911-8. doi:10.1093/jnen/63.9.911
5. Montine TJ, Phelps CH, Beach TG*, et al*. National Institute on Aging-Alzheimer's Association guidelines for the neuropathologic assessment of Alzheimer's disease: a practical approach. *Acta Neuropathol*. Jan 2012;123(1):1-11. doi:10.1007/s00401-011-0910-3
6. Attems J, Toledo JB, Walker L*, et al*. Neuropathological consensus criteria for the evaluation of Lewy pathology in post-mortem brains: a multi-centre study. *Acta Neuropathol*. Feb 2021;141(2):159-172. doi:10.1007/s00401-020-02255-2
7. Braak H, Del Tredici K, Rub U, de Vos RA, Jansen Steur EN, Braak E. Staging of brain pathology related to sporadic Parkinson's disease. *Neurobiol Aging*. Mar-Apr 2003;24(2):197-211. doi:10.1016/s0197-4580(02)00065-9
8. Nelson PT, Dickson DW, Trojanowski JQ*, et al*. Limbic-predominant age-related TDP-43 encephalopathy (LATE): consensus working group report. *Brain*. Jun 1 2019;142(6):1503-1527. doi:10.1093/brain/awz099
9. Skrobot OA, Attems J, Esiri M*, et al*. Vascular cognitive impairment neuropathology guidelines (VCING): the contribution of cerebrovascular pathology to cognitive impairment. *Brain*. Nov 1 2016;139(11):2957-2969. doi:10.1093/brain/aww214

Microglia load: comparisons without corrections

Microglia load was higher in the internal capsule (p=0.043), motor cortex (p=0.036), cerebral peduncle (p=0.040), substantia nigra (p=0.049), red nucleus and superior cerebellar peduncle (p=0.039), and oculomotor complex (p=0.011), between group 1 and 2 cases. Microglial load was higher in the globus pallidus (p=0.037), internal capsule (p=0.008), putamen (p=0.003), dorsomedial thalamus (p=0.005) and substantia nigra (p=0.016) in group 1 versus group 3 cases.

CD20, CD3, CD4, and CD8 cytotoxic cell counts: comparisons without corrections

CD20 positive B cells and CD4 positive T cell densities were similar between groups. CD3 positive T cell densities were higher in the motor area white matter in group 3 compared to group 2. In the motor cortex CD8 positive T cell densities were higher in group 3 versus group 1 (p=0.036) and group 2 (p=0.007), and in the white matter (p=0.036 for Groups 3 and 1; p=0.040 for group 3 versus 2). In the red nucleus/superior cerebellar peduncle CD8 positive T cell densities were higher in group 1 compared to group 2 (p=0.048). Finally, in the oculomotor complex group 3 showed higher CD8 positive T cell densities than group 2 (p=0.005).

p-Tau cytopathologies: comparisons without corrections

P-tau cytopathology scores were compared between groups in cases with PSP stage ≥ 3. Mean oligodendrocytic (*p*=0.001) and astrocytic (*p*=0.032) scores were higher in the motor cortex, neuronal scores higher in the red nucleus (*p*=0.029), and oligodendrocytic scores higher (*p*=0.012) in the cerebral peduncle in group 1 compared to group 2. The neuronal p-tau cytopathology score was higher in the putamen (*p*=0.016) in group 2. Mean neuronal scores were higher in the dorsomedial thalamic nucleus (*p*=0.003) and oligodendrocytic scores were higher in the oculomotor complex (*p*=0.018) in group 1 versus group 3 cases, while neuronal scores were higher in the oculomotor complex (*p*=0.003) and astrocytic scores higher in the motor cortex (*p*=0.039) in group 3 cases. Finally, oligodendrocytic (*p*=0.009) and astrocytic (*p*=0.016) p-tau cytopathology scores were higher in the motor cortex and neuronal scores higher in the oculomotor complex (*p*=0.034) in group 3 versus 2, while neuronal scores in the dorsomedial thalamic nucleus (*p*=0.02) and astrocytic p-tau scores in the oculomotor complex (*p*=0.002) were higher in group 2 versus 3 cases.

Differences in ratios of neuropathological variables: comparisons without corrections

Comparison of ratios of neuropathological variables revealed higher values in group 1 compared to 3 in the putamen and internal capsule for microglia/CD8 (*p*=0.003 and 0.004, respectively) and microglia/CD4 (*p*=0.003 and 0.026, respectively); furthermore in the motor cortex (*p*=0.037), motor area white matter (*p*=0.008), and dorsomedial nucleus of the thalamus (*p*=0.014), and oculomotor nucleus (*p*=0.024) for microglia/CD8. Higher values were observed in group 1 compared to 2 in the putamen and internal capsule for microglia/CD8 (*p*=0.014 and 0.041, respectively) and microglia/CD4 (*p*=0.005 and p=0.022, respectively). CD20/CD8 in the putamen (*p*=0.045) and AT8/CD8 in the red nucleus/superior cerebellar peduncle (*p*=0.026) were higher in group 2 compared to 1. CD8/CD4 was higher in group 3 than 2 in the putamen (*p*=0.031), globus pallidus (*p*=0.008), and motor cortex (p=0.012); furthermore, in the putamen between group 3 and 1 (3 > 1; *p*=0.030). Finally, microglia/CD8 was higher in the motor cortex in group 2 versus 3 (*p*=0.031) and oculomotor complex (*p*=0.042).

Correlation of microglia load and CD8 cell density

CD8 cell density correlated positively with microglia load in the pooled cohort (rho=0.44, p<0.01); this remained significant for pooled regions in each group (rho=0.502, p=<0.01, 0.423, p=<0.01, and 0.751, p=0.007, for groups 1, 2 and 3, respectively) even after correcting for age, ADNC level and PSP stage. Evaluating correlation in HLA groups in different brain regions revealed significance only for a few brain regions (HLA group 2, subthalamic nucleus, rho=0.609, p=0.047; substantia nigra, rho= - 0.609, p=0.047; HLA group 3, dorsomedial thalamic nucleus rho= 0.857, p=0.014; red nucleus, rho= 0.810, p=0.015); significance was lost when correcting for multiple variables (p>0.05).

Table S4. Descriptive statistics of neuropathological variables (AT8: p-tau load: area density; MG: microglia load: area density; CD3: T-cell; CD4: Helper T cell; CD8: cytotoxic T cell; CD20: B cell densities/µm2) in different brain regions listed from highest to lowest in distinct groups of cases (all PSP stage > 2) defined based on HLA haplotype groups (HLA 1, 2, and 3. HLA 4* represents a single case only: *DRB1**10:01-*DQB1**05:01; see manuscript). Color coding indicates the different brain regions for comparability. Abbreviations: GP, globus pallidus; CI, capsula interna; PUT, putamen; MCX, motor cortex; MWM, motor white matter; TDM, thalamus dorsomedial nucleus; STN, subthalamic nucleus; RN, red nucleus; MP, midbrain peduncles; SN, substantia nigra; OC, oculomotor complex.

		HLA 1					HLA 2					HLA 3					HLA 4*	
		N	Mean	SD	SE		N	Mean	SD	SE		N	Mean	SD	SE		N	Mean
AT8	OC	9	5.29	1.58	0.53	STN	9	5.92	3.95	1.32	OC	7	5.72	3.93	1.48	STN	1	10.17
	STN	7	5.09	3.87	1.46	SN	9	4.46	2.77	0.92	STN	6	5.70	3.05	1.24	MCX	1	7.00
	RN	9	4.39	2.34	0.78	GP	7	4.02	3.86	1.46	MCX	5	3.78	1.12	0.50	RN	1	6.30
	SN	9	3.84	1.01	0.34	OC	8	3.66	3.34	1.18	RN	7	3.76	2.40	0.91	OC	1	6.13
	MCX	8	3.62	2.03	0.72	MCX	6	3.34	3.83	1.56	SN	7	3.17	1.71	0.65	GP	1	4.59
	GP	9	2.41	1.71	0.57	RN	9	3.07	2.01	0.67	GP	6	2.44	2.01	0.82	SN	1	3.91
	CI	9	2.23	1.70	0.57	TDM	9	2.47	2.39	0.80	CI	6	2.28	1.81	0.74	MWM	1	2.57
	MWM	8	2.08	1.79	0.63	PUT	9	2.40	1.81	0.60	MWM	5	2.23	0.86	0.38	CI	1	2.53
	MP	9	1.66	0.67	0.22	CI	9	2.15	2.05	0.68	PUT	7	1.69	1.61	0.61	TDM	1	2.37
	TDM	9	1.61	0.82	0.27	MWM	6	1.61	1.45	0.59	MP	7	1.56	1.63	0.62	PUT	1	1.70
	PUT	9	1.26	1.25	0.42	MP	9	0.95	0.59	0.20	TDM	7	1.34	1.02	0.39	MP	1	0.99
MG	RN	9	8.66	2.44	0.81	GP	7	5.80	3.50	1.32	RN	7	5.86	3.83	1.45	RN	1	6.59
	SN	9	7.83	2.51	0.84	SN	9	5.62	3.45	1.15	SN	7	5.19	2.00	0.76	CI	1	5.64
	STN	8	7.82	1.83	0.65	STN	9	5.51	3.70	1.23	STN	6	4.71	2.56	1.05	STN	1	5.37
	CI	9	7.79	1.35	0.45	CI	9	4.55	3.39	1.13	OC	7	4.71	2.99	1.13	GP	1	5.27
	GP	9	7.15	1.97	0.66	RN	9	4.18	3.42	1.14	MP	7	4.35	2.28	0.86	SN	1	5.09
	MWM	8	7.06	2.30	0.81	MWM	6	4.16	2.30	0.94	MWM	5	4.32	1.26	0.56	MP	1	4.04
	MP	9	6.86	2.23	0.74	MP	9	3.81	2.51	0.84	CI	6	3.66	2.36	0.96	OC	1	2.09
	OC	9	6.82	2.64	0.88	OC	8	2.97	2.19	0.77	GP	6	3.60	2.55	1.04	MWM	1	1.84
	TDM	9	4.33	1.70	0.57	TDM	9	2.78	2.05	0.68	MCX	5	1.87	0.56	0.25	PUT	1	1.80
	PUT	9	4.08	1.41	0.47	PUT	9	2.30	1.99	0.66	TDM	7	1.74	1.05	0.40	MCX	1	1.31
	MCX	8	3.81	1.59	0.56	MCX	6	1.59	0.94	0.38	PUT	7	1.33	0.83	0.31	TDM	1	1.05
CD3	SN	9	19.82	12.16	4.05	SN	9	21.34	14.62	4.87	RN	7	30.84	29.99	11.34	STN	1	32.42
	RN	9	19.34	11.39	3.80	GP	7	14.50	8.60	3.25	SN	7	23.65	7.26	2.74	RN	1	20.25
	STN	8	19.08	22.13	7.82	STN	9	12.65	14.58	4.86	MWM	5	16.48	9.15	4.09	SN	1	15.69
	GP	9	12.01	12.35	4.12	RN	9	10.75	13.70	4.57	STN	6	15.61	12.07	4.93	GP	1	11.36
	MWM	8	7.66	6.47	2.29	PUT	9	9.76	3.97	1.32	GP	6	11.59	8.89	3.63	MP	1	9.25
	MP	9	7.06	4.50	1.50	OC	8	5.40	4.85	1.72	OC	7	11.14	7.82	2.95	TDM	1	8.66
	OC	9	6.42	3.65	1.22	MWM	6	4.96	4.06	1.66	MP	7	8.55	6.27	2.37	MWM	1	8.37
	PUT	9	6.42	4.89	1.63	CI	9	4.27	3.83	1.28	PUT	7	7.64	2.72	1.03	OC	1	4.25
	CI	9	4.91	2.58	0.86	MP	9	3.27	3.91	1.30	CI	6	6.28	3.75	1.53	CI	1	3.53
	MCX	8	4.57	5.18	1.83	TDM	9	3.15	3.38	1.13	MCX	5	3.39	0.56	0.25	PUT	1	3.06
	TDM	9	3.06	2.73	0.91	MCX	6	2.58	1.72	0.70	TDM	7	3.06	1.89	0.72	MCX	1	1.29
CD4	SN	9	21.47	15.69	5.23	SN	9	18.03	18.63	6.21	SN	7	20.00	10.90	4.12	SN	1	22.76
	STN	8	9.83	8.74	3.09	GP	7	11.53	9.88	3.73	RN	7	8.65	9.17	3.47	STN	1	12.55
	RN	9	8.33	6.04	2.01	STN	9	10.90	13.35	4.45	OC	7	6.89	6.81	2.57	MWM	1	8.18
	GP	9	6.15	3.32	1.11	PUT	9	7.44	5.69	1.90	MWM	5	6.88	5.97	2.67	GP	1	7.32
	PUT	9	5.40	2.77	0.92	TDM	9	6.24	9.89	3.30	STN	6	6.51	5.52	2.25	RN	1	7.26
	OC	9	5.09	4.76	1.59	RN	9	6.17	5.99	2.00	GP	6	4.76	2.97	1.21	MP	1	5.31
	MWM	8	4.09	2.47	0.87	CI	9	5.59	5.44	1.81	PUT	7	3.96	2.01	0.76	PUT	1	2.35
	MP	9	3.68	2.14	0.71	OC	8	5.58	10.06	3.56	MP	7	3.35	2.57	0.97	OC	1	2.13
	CI	9	3.34	2.43	0.81	MWM	6	5.02	3.56	1.45	CI	6	2.36	1.40	0.57	TDM	1	1.41
	TDM	9	3.10	2.48	0.83	MP	9	2.77	3.05	1.02	TDM	7	2.23	2.58	0.98	MCX	1	1.32
	MCX	8	1.77	1.04	0.37	MCX	6	2.66	2.38	0.97	MCX	5	1.15	0.75	0.33	CI	1	1.17
CD8	STN	8	26.48	28.97	10.24	STN	9	31.65	46.06	15.35	RN	7	41.46	37.10	14.02	STN	1	109.13
	RN	9	25.46	15.30	5.10	SN	9	14.98	10.96	3.65	STN	6	26.16	23.30	9.51	RN	1	49.43
	SN	9	15.01	8.53	2.84	RN	9	13.77	19.66	6.55	MWM	5	23.83	15.87	7.10	GP	1	21.73
	MP	9	12.16	6.75	2.25	GP	7	11.45	8.49	3.21	OC	7	19.16	10.10	3.82	SN	1	19.56
	MWM	8	11.13	11.30	4.00	CI	9	7.80	6.26	2.09	MP	7	15.69	9.87	3.73	OC	1	17.71
	GP	9	10.41	10.28	3.43	MP	9	7.39	6.88	2.29	GP	6	14.73	7.53	3.07	MP	1	13.87
	CI	9	6.25	4.80	1.60	MWM	6	7.28	3.69	1.51	SN	7	14.73	9.70	3.67	MWM	1	10.45
	OC	9	6.10	4.50	1.50	OC	8	6.34	8.10	2.86	CI	6	9.04	4.52	1.84	CI	1	8.17
	PUT	9	5.12	5.63	1.88	PUT	9	5.13	4.45	1.48	PUT	7	6.93	3.47	1.31	TDM	1	4.82
	TDM	9	3.62	2.36	0.79	TDM	9	3.82	2.84	0.95	MCX	5	6.04	3.93	1.76	PUT	1	2.77
	MCX	8	2.41	1.98	0.70	MCX	6	1.53	1.07	0.44	TDM	7	4.55	2.77	1.05	MCX	1	1.66
CD20	SN	9	3.04	2.78	0.93	GP	7	3.16	2.28	0.86	SN	7	2.98	1.61	0.61	SN	1	1.58
	GP	9	1.23	1.24	0.41	PUT	9	2.29	1.87	0.62	PUT	7	1.93	1.70	0.64	RN	1	1.29
	STN	8	1.16	2.23	0.79	SN	9	2.11	1.35	0.45	GP	6	1.44	0.83	0.34	MP	1	0.60
	PUT	9	1.10	0.79	0.26	CI	9	1.25	1.69	0.56	RN	7	0.92	1.53	0.58	MWM	1	0.59
	RN	9	0.80	1.27	0.42	OC	8	0.51	0.89	0.32	MWM	5	0.91	1.20	0.54	GP	1	0.46
	MWM	8	0.61	0.48	0.17	STN	9	0.46	0.40	0.13	OC	7	0.59	0.73	0.28	TDM	1	0.24
	MCX	8	0.40	0.28	0.10	MWM	6	0.46	0.41	0.17	MCX	5	0.57	0.58	0.26	MCX	1	0.22
	MP	9	0.33	0.22	0.07	MCX	6	0.32	0.30	0.12	MP	7	0.52	0.54	0.21	PUT	1	0.19
	OC	9	0.32	0.48	0.16	MP	9	0.23	0.23	0.08	STN	6	0.42	0.38	0.15	CI	1	0.11
	TDM	9	0.17	0.12	0.04	TDM	9	0.21	0.14	0.05	TDM	7	0.34	0.46	0.17	STN	1	0.00
	CI	9	0.16	0.16	0.05	RN	9	0.18	0.12	0.04	CI	6	0.33	0.29	0.12	OC	1	0.00

Table S5. Mann-Whitney test results comparing neuropathological variables (AT8: p-tau load: area density; MG: microglia load: area density; CD3: T-cell; CD4: Helper T cell; CD8: cytotoxic T cell; CD20: B cell densities/µm2) in different brain regions in distinct groups of cases defined based on HLA haplotypes HLA haplotype groups 1, 2, and 3; see manuscript). Red colored box and text indicate p value <0.05 and blue colored text indicates p< 0.01.

Group 1

AT8		MICROGLIA		CD3		CD4		CD8		CD20	
Region	p	Region	p	Region	p	Region	p	Region	p	Region	p
PUT-TDM	0.550	MCx-PUT	0.943	TDM-MCx	0.581	MCx-TDM	0.355	MCx-TDM	0.566	CI-TDM	0.919
PUT-MP	0.427	MCx-TDM	0.732	TDM-CI	0.190	MCx-CI	0.242	MCx-PUT	0.317	CI-OC	0.729
PUT-MWM	0.299	MCx-OC	0.020	TDM-PUT	0.099	MCx-MP	0.136	MCx-CI	0.112	CI-RN	0.308
PUT-CI	0.194	MCx-MP	0.018	TDM-OC	0.061	MCx-MWM	0.093	MCx-OC	0.093	CI-STN	0.274
PUT-GP	0.130	MCx-MWM	0.015	TDM-MWM	0.056	MCx-OC	0.071	MCx-GP	0.022	CI-MP	0.218
PUT-MCx	0.008	MCx-GP	0.008	TDM-MP	0.041	MCx-PUT	0.008	MCx-MWM	0.010	CI-MCx	0.143
PUT-STN	0.003	MCx-SN	0.002	TDM-GP	0.012	MCx-GP	0.005	MCx-MP	0.001	CI-MWM	0.043
PUT-SN	0.001	MCx-CI	0.001	TDM-STN	0.001	MCx-STN	0.003	MCx-SN	0.000	CI-GP	0.005
PUT-RN	0.001	MCx-STN	0.002	TDM-SN	0.000	MCx-RN	0.001	MCx-STN	0.000	CI-PUT	0.001
PUT-OC	0.000	MCx-RN	0.000	TDM-RN	0.000	MCx-SN	0.000	MCx-RN	0.000	CI-SN	0.000
TDM-MP	0.844	PUT-TDM	0.780	MCx-CI	0.471	TDM-CI	0.800	TDM-PUT	0.660	TDM-OC	0.806
TDM-MWM	0.647	PUT-OC	0.020	MCx-PUT	0.294	TDM-MP	0.559	TDM-CI	0.294	TDM-RN	0.358
TDM-CI	0.483	PUT-MP	0.018	MCx-OC	0.207	TDM-MWM	0.422	TDM-OC	0.253	TDM-STN	0.319
TDM-GP	0.360	PUT-MWM	0.015	MCx-MWM	0.187	TDM-OC	0.365	TDM-GP	0.077	TDM-MP	0.258
TDM-MCx	0.037	PUT-GP	0.008	MCx-MP	0.154	TDM-PUT	0.078	TDM-MWM	0.037	TDM-MCx	0.172
TDM-STN	0.018	PUT-SN	0.002	MCx-GP	0.058	TDM-GP	0.050	TDM-MP	0.007	TDM-MWM	0.054
TDM-SN	0.007	PUT-CI	0.001	MCx-STN	0.005	TDM-STN	0.030	TDM-SN	0.003	TDM-GP	0.007
TDM-RN	0.007	PUT-STN	0.001	MCx-SN	0.001	TDM-RN	0.017	TDM-STN	0.002	TDM-PUT	0.002
TDM-OC	0.000	PUT-RN	0.000	MCx-RN	0.000	TDM-SN	0.000	TDM-RN	0.000	TDM-SN	0.000
MP-MWM	0.789	TDM-OC	0.041	CI-PUT	0.735	CI-MP	0.741	PUT-CI	0.542	OC-RN	0.501
MP-CI	0.614	TDM-MP	0.037	CI-OC	0.577	CI-MWM	0.578	PUT-OC	0.483	OC-STN	0.449
MP-GP	0.473	TDM-MWM	0.030	CI-MWM	0.524	CI-OC	0.515	PUT-GP	0.184	OC-MP	0.376
MP-MCx	0.057	TDM-GP	0.018	CI-MP	0.467	CI-PUT	0.132	PUT-MWM	0.097	OC-MCx	0.260
MP-STN	0.029	TDM-SN	0.005	CI-GP	0.226	CI-GP	0.087	PUT-MP	0.023	OC-MWM	0.091
MP-SN	0.013	TDM-CI	0.003	CI-STN	0.031	CI-STN	0.054	PUT-SN	0.010	OC-GP	0.014
MP-RN	0.012	TDM-STN	0.004	CI-SN	0.005	CI-RN	0.033	PUT-STN	0.009	OC-PUT	0.004
MP-OC	0.001	TDM-RN	0.000	CI-RN	0.002	CI-SN	0.000	PUT-RN	0.000	OC-SN	0.001
MWM-CI	0.824	OC-MP	0.973	PUT-OC	0.826	MP-MWM	0.813	CI-OC	0.926	RN-STN	0.917
MWM-GP	0.668	OC-MWM	0.855	PUT-MWM	0.757	MP-OC	0.748	CI-GP	0.472	RN-MP	0.832
MWM-MCx	0.113	OC-GP	0.748	PUT-MP	0.697	MP-PUT	0.240	CI-MWM	0.285	RN-MCx	0.635
MWM-STN	0.061	OC-SN	0.451	PUT-GP	0.383	MP-GP	0.168	CI-MP	0.096	RN-MWM	0.299
MWM-SN	0.032	OC-CI	0.365	PUT-STN	0.067	MP-STN	0.109	CI-SN	0.051	RN-GP	0.073
MWM-RN	0.031	OC-STN	0.352	PUT-SN	0.014	MP-RN	0.072	CI-STN	0.043	RN-PUT	0.028
MWM-OC	0.002	OC-RN	0.104	PUT-RN	0.006	MP-SN	0.001	CI-RN	0.002	RN-SN	0.006
CI-GP	0.831	MP-MWM	0.881	OC-MWM	0.924	MWM-OC	0.939	OC-GP	0.531	STN-MP	0.920
CI-MCx	0.158	MP-GP	0.774	OC-MP	0.866	MWM-PUT	0.365	OC-MWM	0.328	STN-MCx	0.719
CI-STN	0.087	MP-SN	0.472	OC-GP	0.515	MWM-GP	0.270	OC-MP	0.116	STN-MWM	0.364
CI-SN	0.047	MP-CI	0.383	OC-STN	0.105	MWM-STN	0.184	OC-SN	0.063	STN-GP	0.102
CI-RN	0.045	MP-STN	0.369	OC-SN	0.025	MWM-RN	0.130	OC-STN	0.054	STN-PUT	0.043
CI-OC	0.003	MP-RN	0.112	OC-RN	0.011	MWM-SN	0.004	OC-RN	0.003	STN-SN	0.010
GP-MCx	0.229	MWM-GP	0.897	MWM-MP	0.945	OC-PUT	0.393	GP-MWM	0.711	MP-MCx	0.788
GP-STN	0.131	MWM-SN	0.584	MWM-GP	0.592	OC-GP	0.290	GP-MP	0.343	MP-MWM	0.405
GP-SN	0.077	MWM-CI	0.487	MWM-STN	0.139	OC-STN	0.197	GP-SN	0.217	MP-GP	0.113
GP-RN	0.074	MWM-STN	0.467	MWM-SN	0.038	OC-RN	0.139	GP-STN	0.186	MP-PUT	0.048
GP-OC	0.007	MWM-RN	0.163	MWM-RN	0.017	OC-SN	0.004	GP-RN	0.021	MP-SN	0.011
MCx-STN	0.733	GP-SN	0.666	MP-GP	0.630	PUT-GP	0.839	MWM-MP	0.583	MCx-MWM	0.584
MCx-SN	0.608	GP-CI	0.559	MP-STN	0.146	PUT-STN	0.644	MWM-SN	0.408	MCx-GP	0.205
MCx-RN	0.596	GP-STN	0.536	MP-SN	0.038	PUT-RN	0.531	MWM-STN	0.355	MCx-PUT	0.098
MCx-OC	0.154	GP-RN	0.193	MP-RN	0.017	PUT-SN	0.045	MWM-RN	0.061	MCx-SN	0.028
STN-SN	0.886	SN-CI	0.879	GP-STN	0.324	GP-STN	0.791	MP-SN	0.774	MWM-GP	0.482
STN-RN	0.873	SN-STN	0.841	GP-SN	0.112	GP-RN	0.672	MP-STN	0.688	MWM-PUT	0.276
STN-OC	0.306	SN-RN	0.383	GP-RN	0.057	GP-SN	0.072	MP-RN	0.173	MWM-SN	0.102
SN-RN	0.986	CI-STN	0.958	STN-SN	0.578	STN-RN	0.884	SN-STN	0.902	GP-PUT	0.691
SN-OC	0.347	CI-RN	0.472	STN-RN	0.390	STN-SN	0.138	SN-RN	0.283	GP-SN	0.337
RN-OC	0.356	STN-RN	0.519	SN-RN	0.754	RN-SN	0.168	STN-RN	0.358	PUT-SN	0.573

Group 2

AT8		MICROGLIA		CD3		CD4		CD8		CD20	
Region	p	Region	p	Region	p	Region	p	Region	p	Region	p
MP-MWM	0.550	MCx-PUT	0.768	MCx-TDM	0.929	MCx-MP	0.939	MCx-TDM	0.174	RN-TDM	0.745
MP-CI	0.325	MCx-TDM	0.500	MCx-MP	0.859	MCx-OC	0.816	MCx-OC	0.087	RN-MP	0.725
MP-TDM	0.188	MCx-OC	0.349	MCx-CI	0.534	MCx-TDM	0.696	MCx-PUT	0.075	RN-OC	0.491
MP-PUT	0.146	MCx-MP	0.118	MCx-MWM	0.433	MCx-MWM	0.348	MCx-MP	0.010	RN-MCx	0.434
MP-MCx	0.152	MCx-RN	0.094	MCx-OC	0.294	MCx-CI	0.271	MCx-CI	0.009	RN-MWM	0.232
MP-RN	0.030	MCx-MWM	0.112	MCx-RN	0.119	MCx-RN	0.225	MCx-MWM	0.005	RN-STN	0.139
MP-GP	0.040	MCx-CI	0.073	MCx-STN	0.051	MCx-STN	0.092	MCx-RN	0.001	RN-CI	0.036
MP-OC	0.026	MCx-STN	0.019	MCx-PUT	0.009	MCx-PUT	0.077	MCx-GP	0.001	RN-PUT	0.000
MP-SN	0.002	MCx-SN	0.015	MCx-GP	0.003	MCx-GP	0.018	MCx-SN	0.000	RN-SN	0.000
MP-STN	0.001	MCx-GP	0.016	MCx-SN	0.000	MCx-SN	0.003	MCx-STN	0.000	RN-GP	0.000
MWM-CI	0.778	PUT-TDM	0.672	TDM-MP	0.921	MP-OC	0.860	TDM-OC	0.670	TDM-MP	0.978
MWM-TDM	0.561	PUT-OC	0.471	TDM-CI	0.552	MP-TDM	0.725	TDM-PUT	0.639	TDM-OC	0.709
MWM-PUT	0.483	PUT-MP	0.157	TDM-MWM	0.441	MP-MWM	0.341	TDM-MP	0.176	TDM-MCx	0.623
MWM-MCx	0.446	PUT-RN	0.123	TDM-OC	0.285	MP-CI	0.252	TDM-CI	0.167	TDM-MWM	0.366
MWM-RN	0.178	PUT-MWM	0.147	TDM-RN	0.101	MP-RN	0.203	TDM-MWM	0.091	TDM-STN	0.248
MWM-GP	0.194	PUT-CI	0.093	TDM-STN	0.037	MP-STN	0.073	TDM-RN	0.019	TDM-CI	0.077
MWM-OC	0.154	PUT-STN	0.022	TDM-PUT	0.005	MP-PUT	0.058	TDM-GP	0.019	TDM-PUT	0.000
MWM-SN	0.031	PUT-SN	0.016	TDM-GP	0.001	MP-GP	0.011	TDM-SN	0.002	TDM-SN	0.000
MWM-STN	0.016	PUT-GP	0.019	TDM-SN	0.000	MP-SN	0.001	TDM-STN	0.000	TDM-GP	0.000
CI-TDM	0.739	TDM-OC	0.757	MP-CI	0.620	OC-TDM	0.869	OC-PUT	0.976	MP-OC	0.729
CI-PUT	0.639	TDM-MP	0.321	MP-MWM	0.495	OC-MWM	0.441	OC-MP	0.375	MP-MCx	0.640
CI-MCx	0.580	TDM-RN	0.263	MP-OC	0.331	OC-CI	0.350	OC-CI	0.361	MP-MWM	0.379
CI-RN	0.234	TDM-MWM	0.285	MP-RN	0.123	OC-RN	0.290	OC-MWM	0.205	MP-STN	0.259
CI-GP	0.256	TDM-CI	0.210	MP-STN	0.047	OC-STN	0.117	OC-RN	0.066	MP-CI	0.082
CI-OC	0.201	TDM-STN	0.063	MP-PUT	0.007	OC-PUT	0.097	OC-GP	0.059	MP-PUT	0.000
CI-SN	0.036	TDM-SN	0.048	MP-GP	0.002	OC-GP	0.021	OC-SN	0.011	MP-SN	0.000
CI-STN	0.018	TDM-GP	0.051	MP-SN	0.000	OC-SN	0.003	OC-STN	0.002	MP-GP	0.000
TDM-PUT	0.892	OC-MP	0.514	CI-MWM	0.812	TDM-MWM	0.524	PUT-MP	0.377	OC-MCx	0.885
TDM-MCx	0.799	OC-RN	0.438	CI-OC	0.623	TDM-CI	0.427	PUT-CI	0.362	OC-MWM	0.585
TDM-RN	0.391	OC-MWM	0.444	CI-RN	0.295	TDM-RN	0.357	PUT-MWM	0.204	OC-STN	0.455
TDM-GP	0.410	OC-CI	0.364	CI-STN	0.137	TDM-STN	0.149	PUT-RN	0.062	OC-CI	0.179
TDM-OC	0.340	OC-STN	0.135	CI-PUT	0.027	TDM-PUT	0.123	PUT-GP	0.056	OC-PUT	0.002
TDM-SN	0.077	OC-SN	0.108	CI-GP	0.008	TDM-GP	0.027	PUT-SN	0.010	OC-SN	0.002
TDM-STN	0.041	OC-GP	0.107	CI-SN	0.001	TDM-SN	0.003	PUT-STN	0.001	OC-GP	0.001
PUT-MCx	0.894	MP-RN	0.899	MWM-OC	0.834	MWM-CI	0.942	MP-CI	0.978	MCx-MWM	0.707
PUT-RN	0.470	MP-MWM	0.856	MWM-RN	0.485	MWM-RN	0.853	MP-MWM	0.631	MCx-STN	0.589
PUT-GP	0.485	MP-CI	0.794	MWM-STN	0.274	MWM-STN	0.513	MP-RN	0.325	MCx-CI	0.276
PUT-OC	0.410	MP-STN	0.386	MWM-PUT	0.082	MWM-PUT	0.458	MP-GP	0.279	MCx-PUT	0.006
PUT-SN	0.102	MP-SN	0.325	MWM-GP	0.029	MWM-GP	0.163	MP-SN	0.088	MCx-SN	0.006
PUT-STN	0.057	MP-GP	0.306	MWM-SN	0.005	MWM-SN	0.046	MP-STN	0.021	MCx-GP	0.004
MCx-RN	0.608	RN-MWM	0.945	OC-RN	0.600	CI-RN	0.899	CI-MWM	0.648	MWM-STN	0.897
MCx-GP	0.613	RN-CI	0.892	OC-STN	0.341	CI-STN	0.516	CI-RN	0.339	MWM-CI	0.498
MCx-OC	0.541	RN-STN	0.459	OC-PUT	0.098	CI-PUT	0.454	CI-GP	0.290	MWM-PUT	0.020
MCx-SN	0.184	RN-SN	0.391	OC-GP	0.034	CI-GP	0.143	CI-SN	0.093	MWM-SN	0.019
MCx-STN	0.117	RN-GP	0.365	OC-SN	0.005	CI-SN	0.032	CI-STN	0.023	MWM-GP	0.014
RN-GP	0.982	MWM-CI	0.958	RN-STN	0.658	RN-STN	0.601	MWM-RN	0.690	STN-CI	0.540
RN-OC	0.902	MWM-STN	0.553	RN-PUT	0.244	RN-PUT	0.534	MWM-GP	0.599	STN-PUT	0.014
RN-SN	0.362	MWM-SN	0.485	RN-GP	0.094	RN-GP	0.178	MWM-SN	0.296	STN-SN	0.013
RN-STN	0.237	MWM-GP	0.450	RN-SN	0.019	RN-SN	0.043	MWM-STN	0.115	STN-GP	0.010
GP-OC	0.925	CI-STN	0.546	STN-PUT	0.470	STN-PUT	0.921	RN-GP	0.871	CI-PUT	0.066
GP-SN	0.407	CI-SN	0.470	STN-GP	0.206	STN-GP	0.391	RN-SN	0.470	CI-SN	0.062
GP-STN	0.279	CI-GP	0.436	STN-SN	0.056	STN-SN	0.134	RN-STN	0.188	CI-GP	0.044
OC-SN	0.447	STN-SN	0.907	PUT-GP	0.556	PUT-GP	0.445	GP-SN	0.608	PUT-SN	0.978
OC-STN	0.306	STN-GP	0.831	PUT-SN	0.234	PUT-SN	0.162	GP-STN	0.285	PUT-GP	0.772
SN-STN	0.787	SN-GP	0.917	GP-SN	0.599	GP-SN	0.587	SN-STN	0.552	SN-GP	0.792

Group 3

AT8		MICROGLIA		CD3		CD4		CD8		CD20	
Region	p	Region	p	Region	p	Region	p	Region	p	Region	p
TDM-MP	0.906	PUT-TDM	0.646	TDM-MCx	0.824	MCx-TDM	0.484	TDM-MCx	0.657	TDM-CI	0.861
TDM-PUT	0.783	PUT-MCx	0.583	TDM-CI	0.309	MCx-CI	0.290	TDM-PUT	0.431	TDM-OC	0.554
TDM-CI	0.363	PUT-GP	0.116	TDM-PUT	0.115	MCx-MP	0.118	TDM-CI	0.207	TDM-STN	0.513
TDM-MWM	0.376	PUT-CI	0.058	TDM-MP	0.112	MCx-PUT	0.029	TDM-SN	0.022	TDM-MP	0.400
TDM-GP	0.284	PUT-MP	0.015	TDM-OC	0.040	MCx-GP	0.021	TDM-GP	0.025	TDM-RN	0.328
TDM-SN	0.093	PUT-MWM	0.024	TDM-GP	0.044	MCx-OC	0.012	TDM-MP	0.018	TDM-MCx	0.366
TDM-RN	0.038	PUT-OC	0.013	TDM-STN	0.014	MCx-STN	0.012	TDM-STN	0.004	TDM-MWM	0.197
TDM-MCx	0.038	PUT-STN	0.014	TDM-MWM	0.006	MCx-MWM	0.010	TDM-OC	0.003	TDM-GP	0.008
TDM-OC	0.007	PUT-RN	0.003	TDM-RN	0.002	MCx-RN	0.004	TDM-MWM	0.003	TDM-PUT	0.004
TDM-STN	0.005	PUT-SN	0.002	TDM-SN	0.000	MCx-SN	0.000	TDM-RN	0.001	TDM-SN	0.000
MP-PUT	0.875	TDM-MCx	0.897	MCx-CI	0.472	TDM-CI	0.679	MCx-PUT	0.783	CI-OC	0.695
MP-CI	0.425	TDM-GP	0.258	MCx-PUT	0.224	TDM-MP	0.344	MCx-CI	0.465	CI-STN	0.645
MP-MWM	0.437	TDM-CI	0.146	MCx-MP	0.220	TDM-PUT	0.103	MCx-SN	0.098	CI-MP	0.527
MP-GP	0.338	TDM-MP	0.047	MCx-OC	0.100	TDM-GP	0.075	MCx-GP	0.102	CI-RN	0.444
MP-SN	0.118	TDM-MWM	0.066	MCx-GP	0.101	TDM-OC	0.047	MCx-MP	0.086	CI-MCx	0.476
MP-RN	0.050	TDM-OC	0.042	MCx-STN	0.042	TDM-STN	0.045	MCx-STN	0.028	CI-MWM	0.277
MP-MCx	0.049	TDM-STN	0.044	MCx-MWM	0.018	TDM-MWM	0.039	MCx-OC	0.021	CI-GP	0.017
MP-OC	0.010	TDM-RN	0.013	MCx-RN	0.010	TDM-RN	0.016	MCx-MWM	0.021	CI-PUT	0.009
MP-STN	0.006	TDM-SN	0.007	MCx-SN	0.000	TDM-SN	0.000	MCx-RN	0.011	CI-SN	0.001
PUT-CI	0.519	MCx-GP	0.361	CI-PUT	0.620	CI-MP	0.621	PUT-CI	0.614	OC-STN	0.931
PUT-MWM	0.527	MCx-CI	0.226	CI-MP	0.611	CI-PUT	0.250	PUT-SN	0.131	OC-MP	0.803
PUT-GP	0.419	MCx-MP	0.093	CI-OC	0.342	CI-GP	0.187	PUT-GP	0.136	OC-RN	0.698
PUT-SN	0.160	MCx-MWM	0.113	CI-GP	0.335	CI-OC	0.136	PUT-MP	0.115	OC-MCx	0.715
PUT-RN	0.072	MCx-OC	0.084	CI-STN	0.169	CI-STN	0.126	PUT-STN	0.036	OC-MWM	0.453
PUT-MCx	0.068	MCx-STN	0.084	CI-MWM	0.081	CI-MWM	0.105	PUT-OC	0.026	OC-GP	0.037
PUT-OC	0.016	MCx-RN	0.032	CI-RN	0.053	CI-RN	0.058	PUT-MWM	0.027	OC-PUT	0.022
PUT-STN	0.010	MCx-SN	0.021	CI-SN	0.003	CI-SN	0.000	PUT-RN	0.014	OC-SN	0.002
CI-MWM	0.985	GP-CI	0.755	PUT-MP	0.990	MP-PUT	0.495	CI-SN	0.344	STN-MP	0.878
CI-GP	0.876	GP-MP	0.439	PUT-OC	0.636	MP-GP	0.382	CI-GP	0.342	STN-RN	0.775
CI-SN	0.481	GP-MWM	0.459	PUT-GP	0.614	MP-OC	0.300	CI-MP	0.313	STN-MCx	0.785
CI-RN	0.279	GP-OC	0.410	PUT-STN	0.352	MP-STN	0.273	CI-STN	0.126	STN-MWM	0.518
CI-MCx	0.242	GP-STN	0.395	PUT-MWM	0.182	MP-MWM	0.228	CI-OC	0.104	STN-GP	0.054
CI-OC	0.094	GP-RN	0.206	PUT-RN	0.134	MP-RN	0.145	CI-MWM	0.095	STN-PUT	0.034
CI-STN	0.063	GP-SN	0.149	PUT-SN	0.011	MP-SN	0.001	CI-RN	0.062	STN-SN	0.003
MWM-GP	0.897	CI-MP	0.653	MP-OC	0.646	PUT-GP	0.827	SN-GP	0.968	MP-RN	0.890
MWM-SN	0.516	CI-MWM	0.657	MP-GP	0.623	PUT-OC	0.723	SN-MP	0.948	MP-MCx	0.891
MWM-RN	0.313	CI-OC	0.617	MP-STN	0.358	PUT-STN	0.660	SN-STN	0.520	MP-MWM	0.601
MWM-MCx	0.270	CI-STN	0.590	MP-MWM	0.186	PUT-MWM	0.560	SN-OC	0.478	MP-GP	0.064
MWM-OC	0.116	CI-RN	0.346	MP-RN	0.138	PUT-RN	0.438	SN-MWM	0.407	MP-PUT	0.040
MWM-STN	0.080	CI-SN	0.263	MP-SN	0.012	PUT-SN	0.011	SN-RN	0.338	MP-SN	0.004
GP-SN	0.587	MP-MWM	0.975	OC-GP	0.960	GP-OC	0.903	GP-MP	0.982	RN-MCx	0.991
GP-RN	0.357	MP-OC	0.958	OC-STN	0.633	GP-STN	0.832	GP-STN	0.561	RN-MWM	0.691
GP-MCx	0.307	MP-STN	0.913	OC-MWM	0.366	GP-MWM	0.717	GP-OC	0.521	RN-GP	0.086
GP-OC	0.130	MP-RN	0.609	OC-RN	0.306	GP-RN	0.599	GP-MWM	0.444	RN-PUT	0.056
GP-STN	0.089	MP-SN	0.486	OC-SN	0.039	GP-SN	0.026	GP-RN	0.378	RN-SN	0.006
SN-RN	0.694	MWM-OC	0.987	GP-STN	0.681	OC-STN	0.921	MP-STN	0.562	MCx-MWM	0.721
SN-MCx	0.588	MWM-STN	0.944	GP-MWM	0.408	OC-MWM	0.796	MP-OC	0.520	MCx-GP	0.117
SN-OC	0.312	MWM-RN	0.663	GP-RN	0.350	OC-RN	0.674	MP-MWM	0.442	MCx-PUT	0.083
SN-STN	0.221	MWM-SN	0.546	GP-SN	0.054	OC-SN	0.028	MP-RN	0.372	MCx-SN	0.012
RN-MCx	0.855	OC-STN	0.953	STN-MWM	0.663	STN-MWM	0.873	STN-OC	0.970	MWM-GP	0.233
RN-OC	0.537	OC-RN	0.646	STN-RN	0.612	STN-RN	0.760	STN-MWM	0.833	MWM-PUT	0.178
RN-STN	0.398	OC-SN	0.520	STN-SN	0.133	STN-SN	0.045	STN-RN	0.781	MWM-SN	0.034
MCx-OC	0.703	STN-RN	0.702	MWM-RN	0.975	MWM-RN	0.901	OC-MWM	0.855	GP-PUT	0.905
MCx-STN	0.548	STN-SN	0.576	MWM-SN	0.328	MWM-SN	0.081	OC-RN	0.803	GP-SN	0.354
OC-STN	0.801	RN-SN	0.854	RN-SN	0.300	RN-SN	0.076	MWM-RN	0.964	PUT-SN	0.400

Table S6. Descriptive statistics of ratios of neuropathological variables (AT8: p-tau load: area density; MG: microglia load: area density; CD3: T-cell; CD4: Helper T cell; CD8: cytotoxic T cell; CD20: B cell densities/μm2) in different brain regions in distinct groups of cases (all PSP stage > 2) defined based on HLA haplotypes HLA haplotypes (HLA 1, 2, and 3. HLA 4* represents a single case only: *DRB1**10:01-*DQB1**05:01; see manuscript). Color coding indicates the different brain regions for comparability. Abbreviations: GP, globus pallidus; CI, capsula interna; PUT, putamen; MCX, motor cortex; MWM, motor white matter; TDM, thalamus dorsomedial nucleus; STN, subthalamic nucleus; RN, red nucleus; MP, midbrain peduncles; SN, substantia nigra; OC, oculomotor complex.

		HLA 1					HLA 2					HLA 3					HLA 4*	
		N	Mean	SD	SE		N	Mean	SD	SE		N	Mean	SD	SE		N	Mean
CD20/CD8	PUT	9	0.27	0.11	0.04	PUT	9	0.54	0.27	0.09	SN	7	1.51	3.47	1.31	RN	1	0.9749
	MCX	8	0.26	0.23	0.08	GP	6	0.27	0.12	0.05	PUT	6	0.23	0.13	0.05	MCX	1	0.1319
	SN	9	0.21	0.11	0.04	CI	8	0.22	0.33	0.12	MP	7	0.14	0.28	0.11	SN	1	0.0808
	GP	9	0.16	0.11	0.04	SN	9	0.21	0.13	0.04	MCX	5	0.13	0.13	0.06	PUT	1	0.0689
	STN	8	0.06	0.05	0.02	MCX	6	0.19	0.10	0.04	GP	6	0.11	0.05	0.02	MWM	1	0.0568
	MWM	8	0.06	0.03	0.01	MWM	6	0.09	0.10	0.04	RN	7	0.10	0.16	0.06	TDM	1	0.0501
	TDM	9	0.06	0.05	0.02	RN	9	0.09	0.14	0.05	STN	6	0.09	0.09	0.04	MP	1	0.0430
	OC	8	0.05	0.06	0.02	OC	8	0.09	0.11	0.04	TDM	7	0.09	0.08	0.03	GP	1	0.0211
	RN	9	0.04	0.05	0.02	TDM	9	0.08	0.05	0.02	MWM	5	0.05	0.06	0.03	CI	1	0.0136
	MP	9	0.03	0.02	0.01	MP	9	0.05	0.06	0.02	OC	7	0.04	0.08	0.03	STN	1	0.0000
	CI	9	0.03	0.02	0.01	STN	9	0.05	0.05	0.02	CI	5	0.04	0.04	0.02	OC	1	0.0000
AT8/CD8	MCX	8	3.52	4.46	1.58	MCX	6	1.82	1.94	0.79	MCX	5	0.89	0.77	0.34	MCX	1	4.2124
	OC	8	0.87	1.06	0.38	OC	8	1.14	1.79	0.63	CI	5	0.37	0.45	0.20	PUT	1	0.6127
	TDM	9	0.53	0.40	0.13	PUT	9	0.77	0.71	0.24	SN	7	0.34	0.28	0.11	RN	1	0.5615
	CI	9	0.50	0.55	0.18	RN	9	0.73	0.53	0.18	STN	6	0.33	0.40	0.16	TDM	1	0.4921
	SN	9	0.46	0.51	0.17	SN	9	0.59	0.48	0.16	RN	7	0.33	0.54	0.20	OC	1	0.3460
	STN	8	0.43	0.57	0.20	TDM	9	0.57	0.48	0.16	OC	7	0.32	0.27	0.10	CI	1	0.3098
	PUT	9	0.43	0.39	0.13	GP	6	0.53	0.63	0.26	TDM	7	0.31	0.26	0.10	MWM	1	0.2456
	GP	9	0.34	0.36	0.12	CI	8	0.39	0.31	0.11	PUT	6	0.28	0.33	0.13	GP	1	0.2113
	MWM	8	0.33	0.33	0.12	STN	9	0.36	0.47	0.16	GP	6	0.23	0.26	0.11	SN	1	0.1999
	MP	9	0.27	0.27	0.09	MP	9	0.27	0.32	0.11	MP	7	0.17	0.21	0.08	STN	1	0.0932
	RN	9	0.23	0.23	0.08	MWM	6	0.15	0.13	0.05	MWM	5	0.15	0.14	0.06	MP	1	0.0711
AT8/CD4	OC	8	2.56	2.88	1.02	OC	8	3.45	5.83	2.06	MCX	5	3.55	3.06	1.37	MCX	1	5.3213
	MCX	8	2.25	1.24	0.44	TDM	9	2.47	3.33	1.11	TDM	7	1.64	2.12	0.80	OC	1	2.8810
	TDM	9	1.40	1.85	0.62	RN	9	2.24	2.39	0.80	OC	7	1.32	1.56	0.59	CI	1	2.1665
	CI	9	1.01	1.01	0.34	MP	9	1.51	2.22	0.74	CI	5	0.87	0.49	0.22	TDM	1	1.6789
	MP	9	0.99	1.22	0.41	CI	8	1.46	2.34	0.83	STN	6	0.77	0.75	0.30	STN	1	0.8100
	RN	9	0.90	1.06	0.35	MCX	6	1.14	0.69	0.28	GP	6	0.72	0.64	0.26	PUT	1	0.7217
	MWM	8	0.70	0.82	0.29	MWM	6	1.02	1.95	0.80	MWM	5	0.59	0.45	0.20	RN	1	0.6752
	GP	9	0.52	0.45	0.15	STN	9	0.89	1.19	0.40	RN	7	0.57	0.40	0.15	GP	1	0.6274
	STN	8	0.50	0.59	0.21	SN	9	0.77	1.03	0.34	MP	7	0.51	0.44	0.17	MWM	1	0.3135
	SN	9	0.39	0.29	0.10	PUT	9	0.64	0.68	0.23	PUT	6	0.45	0.42	0.17	MP	1	0.1857
	PUT	9	0.34	0.55	0.18	GP	6	0.36	0.40	0.16	SN	7	0.19	0.17	0.07	SN	1	0.1718
MG/CD8	MCX	8	2.92	2.69	0.95	MCX	6	1.89	2.00	0.82	MP	7	1.74	3.91	1.48	MCX	1	0.7870
	CI	9	1.84	1.03	0.34	RN	9	1.24	1.52	0.51	SN	7	1.03	1.74	0.66	CI	1	0.6903
	PUT	9	1.38	0.90	0.30	TDM	9	1.18	1.89	0.63	STN	6	0.76	1.05	0.43	PUT	1	0.6475
	TDM	9	1.23	0.80	0.27	GP	6	1.06	1.43	0.58	RN	7	0.74	1.40	0.53	RN	1	0.6249
	MWM	8	1.20	0.97	0.34	OC	8	0.99	0.95	0.33	CI	5	0.56	0.69	0.31	MP	1	0.2912
	OC	8	1.11	1.38	0.49	PUT	9	0.93	1.75	0.58	TDM	7	0.42	0.20	0.07	SN	1	0.2604
	MP	9	0.98	0.70	0.23	MP	9	0.91	0.99	0.33	MCX	5	0.41	0.22	0.10	GP	1	0.2424
	GP	9	0.89	0.96	0.32	SN	9	0.84	0.77	0.26	GP	6	0.30	0.37	0.15	TDM	1	0.2173
	STN	8	0.88	1.24	0.44	CI	8	0.79	0.69	0.24	OC	7	0.25	0.13	0.05	MWM	1	0.1758
	SN	9	0.79	0.78	0.26	MWM	6	0.63	0.38	0.16	MWM	5	0.24	0.15	0.07	OC	1	0.1183
	RN	9	0.46	0.41	0.14	STN	9	0.41	0.55	0.18	PUT	6	0.21	0.17	0.07	STN	1	0.0492
MG/CD4	CI	9	3.50	2.28	0.76	TDM	9	3.27	7.02	2.34	MP	7	2.12	2.44	0.92	CI	1	4.8268
	OC	8	3.01	3.44	1.22	MP	9	3.04	3.51	1.17	TDM	7	1.65	1.63	0.61	MCX	1	0.9942
	MP	9	3.01	2.21	0.74	MWM	6	2.27	3.67	1.50	MCX	5	1.56	0.82	0.37	OC	1	0.9846
	TDM	9	2.45	1.65	0.55	OC	8	1.99	1.89	0.67	STN	6	1.37	1.38	0.56	PUT	1	0.7627
	MWM	8	2.32	1.50	0.53	RN	9	1.86	1.39	0.46	CI	5	1.30	0.50	0.23	MP	1	0.7601
	MCX	8	2.16	1.25	0.44	CI	8	1.71	2.07	0.73	MWM	5	0.99	0.76	0.34	RN	1	0.7515
	RN	9	1.35	0.88	0.29	MCX	6	1.12	0.77	0.31	OC	7	0.96	0.68	0.26	TDM	1	0.7412
	GP	9	1.26	1.03	0.34	STN	9	0.87	0.98	0.33	RN	7	0.89	0.47	0.18	GP	1	0.7198
	STN	8	1.00	1.16	0.41	SN	9	0.69	0.71	0.24	GP	6	0.86	0.76	0.31	STN	1	0.4281
	PUT	9	0.81	0.27	0.09	GP	6	0.59	0.44	0.18	PUT	6	0.33	0.18	0.07	MWM	1	0.2245
	SN	9	0.58	0.41	0.14	PUT	9	0.38	0.26	0.09	SN	7	0.31	0.23	0.09	SN	1	0.2238
CD8/CD4	OC	8	4.09	4.25	1.50	MP	9	6.72	8.36	2.79	MP	7	7.35	8.30	3.14	STN	1	8.6945
	RN	9	4.07	3.36	1.12	TDM	9	6.45	10.75	3.58	MWM	5	5.76	6.16	2.75	OC	1	8.3260
	MP	9	4.02	3.28	1.09	MWM	6	5.65	9.74	3.98	TDM	7	5.14	5.12	1.94	CI	1	6.9925
	MWM	8	3.03	2.57	0.91	RN	9	4.46	4.22	1.41	RN	7	4.98	3.93	1.49	TDM	1	3.4118
	CI	9	2.90	2.69	0.90	STN	9	3.59	3.30	1.10	OC	7	4.24	3.11	1.17	GP	1	2.9689
	TDM	9	2.60	1.59	0.53	CI	8	3.25	4.14	1.46	CI	5	4.18	2.05	0.92	MP	1	2.6101
	STN	8	2.52	2.52	0.89	OC	8	2.98	2.48	0.88	MCX	5	4.13	1.82	0.81	MWM	1	1.2767
	MCX	8	2.30	2.27	0.80	SN	9	1.45	1.29	0.43	GP	6	3.38	1.23	0.50	MCX	1	1.2632
	GP	9	2.02	0.97	0.32	MCX	6	1.29	1.44	0.59	STN	6	3.31	2.14	0.87	RN	1	1.2026
	SN	9	1.25	1.22	0.41	GP	6	1.08	0.53	0.22	PUT	6	1.96	0.72	0.29	PUT	1	1.1779
	PUT	9	0.99	0.77	0.26	PUT	9	0.98	0.43	0.14	SN	7	1.04	1.34	0.51	SN	1	0.8595

Table S7. Mann-Whitney test results comparing ratios of neuropathological variables (AT8: p-tau load: area density; MG: microglia load: area density; CD3: T-cell; CD4: Helper T cell; CD8: cytotoxic T cell; CD20: B cell densities/μm2) in different brain regions in distinct groups of cases defined based on HLA haplotype groups (see manuscript). Red colored box and text indicate p value <0.05 and blue colored text indicates p< 0.01.

ONLINE SUPPLEMENTARY FILE

Group 1											
**CD20/CD8**		**AT8/CD8**		**AT8/CD4**		**MG/CD8**		**MG/CD4**		**CD8/CD4**	
Region	p	Region	p	Region	p	Region	p	Region	p	Region	p
CI-RN	0.861	CI-RN	0.861	PUT-SN	0.407	RN-STN	0.579	SN-PUT	0.578	PUT-SN	0.707
CI-MP	0.672	CI-MP	0.672	PUT-STN	0.360	RN-GP	0.385	SN-STN	0.583	PUT-MCx	0.200
CI-OC	0.491	CI-OC	0.491	PUT-GP	0.245	RN-SN	0.381	SN-GP	0.153	PUT-STN	0.099
CI-TDM	0.305	CI-TDM	0.305	PUT-MWM	0.134	RN-OC	0.209	SN-RN	0.107	PUT-GP	0.064
CI-STN	0.189	CI-STN	0.189	PUT-RN	0.100	RN-MP	0.110	SN-OC	0.010	PUT-CI	0.057
CI-MWM	0.140	CI-MWM	0.140	PUT-MP	0.054	RN-MWM	0.070	SN-MCx	0.003	PUT-TDM	0.031
CI-GP	0.002	CI-GP	0.002	PUT-CI	0.030	RN-TDM	0.032	SN-MWM	0.003	PUT-MWM	0.022
CI-SN	0.000	CI-SN	0.000	PUT-TDM	0.012	RN-PUT	0.017	SN-TDM	0.002	PUT-OC	0.020
CI-MCx	0.000	CI-MCx	0.000	PUT-OC	0.003	RN-MCx	0.010	SN-MP	0.000	PUT-RN	0.003
CI-PUT	0.000	CI-PUT	0.000	PUT-MCx	0.000	RN-CI	0.002	SN-CI	0.000	PUT-MP	0.002
RN-MP	0.804	RN-MP	0.804	SN-STN	0.911	STN-GP	0.774	PUT-STN	0.992	SN-MCx	0.359
RN-OC	0.604	RN-OC	0.604	SN-GP	0.739	STN-SN	0.768	PUT-GP	0.383	SN-STN	0.199
RN-TDM	0.395	RN-TDM	0.395	SN-MWM	0.487	STN-OC	0.496	PUT-RN	0.291	SN-GP	0.139
RN-STN	0.253	RN-STN	0.253	SN-RN	0.414	STN-MP	0.319	PUT-OC	0.042	SN-CI	0.126
RN-MWM	0.191	RN-MWM	0.191	SN-MP	0.272	STN-MWM	0.223	PUT-MCx	0.017	SN-TDM	0.074
RN-GP	0.003	RN-GP	0.003	SN-CI	0.179	STN-TDM	0.128	PUT-MWM	0.015	SN-MWM	0.054
RN-SN	0.001	RN-SN	0.001	SN-TDM	0.092	STN-PUT	0.079	PUT-TDM	0.010	SN-OC	0.050
RN-MCx	0.000	RN-MCx	0.000	SN-OC	0.030	STN-MCx	0.050	PUT-MP	0.003	SN-RN	0.010
RN-PUT	0.000	RN-PUT	0.000	SN-MCx	0.001	STN-CI	0.014	PUT-CI	0.001	SN-MP	0.007
MP-OC	0.781	MP-OC	0.781	STN-GP	0.832	GP-SN	0.993	STN-GP	0.403	MCx-STN	0.720
MP-TDM	0.547	MP-TDM	0.547	STN-MWM	0.571	GP-OC	0.679	STN-RN	0.310	MCx-GP	0.604
MP-STN	0.367	MP-STN	0.367	STN-RN	0.496	GP-MP	0.465	STN-OC	0.049	MCx-CI	0.570
MP-MWM	0.286	MP-MWM	0.286	STN-MP	0.340	GP-MWM	0.333	STN-MCx	0.021	MCx-TDM	0.414
MP-GP	0.007	MP-GP	0.007	STN-CI	0.234	GP-TDM	0.203	STN-MWM	0.019	MCx-MWM	0.327
MP-SN	0.002	MP-SN	0.002	STN-TDM	0.128	GP-PUT	0.130	STN-TDM	0.013	MCx-OC	0.312
MP-MCx	0.001	MP-MCx	0.001	STN-OC	0.045	GP-MCx	0.083	STN-MP	0.004	MCx-RN	0.113
MP-PUT	0.000	MP-PUT	0.000	STN-MCx	0.002	GP-CI	0.025	STN-CI	0.001	MCx-MP	0.092
OC-TDM	0.758	OC-TDM	0.758	GP-MWM	0.710	SN-OC	0.686	GP-RN	0.854	STN-GP	0.881
OC-STN	0.543	OC-STN	0.543	GP-RN	0.629	SN-MP	0.470	GP-OC	0.235	STN-CI	0.842
OC-MWM	0.443	OC-MWM	0.443	GP-MP	0.444	SN-MWM	0.337	GP-MCx	0.124	STN-TDM	0.654
OC-GP	0.019	OC-GP	0.019	GP-CI	0.313	SN-TDM	0.206	GP-MWM	0.113	STN-MWM	0.534
OC-SN	0.005	OC-SN	0.005	GP-TDM	0.177	SN-PUT	0.132	GP-TDM	0.090	STN-OC	0.514
OC-MCx	0.004	OC-MCx	0.004	GP-OC	0.064	SN-MCx	0.084	GP-MP	0.034	STN-RN	0.224
OC-PUT	0.000	OC-PUT	0.000	GP-MCx	0.003	SN-CI	0.026	GP-CI	0.012	STN-MP	0.188
TDM-STN	0.751	TDM-STN	0.751	MWM-RN	0.922	OC-MP	0.767	RN-OC	0.312	GP-CI	0.959
TDM-MWM	0.630	TDM-MWM	0.630	MWM-MP	0.710	OC-MWM	0.589	RN-MCx	0.174	GP-TDM	0.758
TDM-GP	0.035	TDM-GP	0.035	MWM-CI	0.543	OC-TDM	0.411	RN-MWM	0.159	GP-MWM	0.625
TDM-SN	0.010	TDM-SN	0.010	MWM-TDM	0.348	OC-PUT	0.291	RN-TDM	0.131	GP-OC	0.602
TDM-MCx	0.008	TDM-MCx	0.008	MWM-OC	0.151	OC-MCx	0.199	RN-MP	0.052	GP-RN	0.272
TDM-PUT	0.001	TDM-PUT	0.001	MWM-MCx	0.011	OC-CI	0.078	RN-CI	0.020	GP-MP	0.230
STN-MWM	0.874	STN-MWM	0.874	RN-MP	0.778	MP-MWM	0.795	OC-MCx	0.734	CI-TDM	0.798
STN-GP	0.085	STN-GP	0.085	RN-CI	0.599	MP-TDM	0.587	OC-MWM	0.700	CI-MWM	0.660
STN-SN	0.030	STN-SN	0.030	RN-TDM	0.385	MP-PUT	0.434	OC-TDM	0.650	CI-OC	0.637
STN-MCx	0.023	STN-MCx	0.023	RN-OC	0.167	MP-MCx	0.306	OC-MP	0.383	CI-RN	0.295
STN-PUT	0.003	STN-PUT	0.003	RN-MCx	0.012	MP-CI	0.131	OC-CI	0.211	CI-MP	0.250
MWM-GP	0.119	MWM-GP	0.119	MP-CI	0.807	MWM-TDM	0.789	MCx-MWM	0.964	TDM-MWM	0.849
MWM-SN	0.045	MWM-SN	0.045	MP-TDM	0.558	MWM-PUT	0.617	MCx-TDM	0.917	TDM-OC	0.823
MWM-MCx	0.034	MWM-MCx	0.034	MP-OC	0.268	MWM-MCx	0.457	MCx-MP	0.601	TDM-RN	0.429
MWM-PUT	0.005	MWM-PUT	0.005	MP-MCx	0.026	MWM-CI	0.228	MCx-CI	0.368	TDM-MP	0.372
GP-SN	0.644	GP-SN	0.644	CI-TDM	0.732	TDM-PUT	0.811	MWM-TDM	0.955	MWM-OC	0.975
GP-MCx	0.532	GP-MCx	0.532	CI-OC	0.384	TDM-MCx	0.619	MWM-MP	0.634	MWM-RN	0.564
GP-PUT	0.194	GP-PUT	0.194	CI-MCx	0.046	TDM-CI	0.334	MWM-CI	0.393	MWM-MP	0.499
SN-MCx	0.860	SN-MCx	0.860	TDM-OC	0.589	PUT-MCx	0.791	TDM-MP	0.666	OC-RN	0.587
SN-PUT	0.402	SN-PUT	0.402	TDM-MCx	0.096	PUT-CI	0.467	TDM-CI	0.412	OC-MP	0.520
MCx-PUT	0.524	MCx-PUT	0.524	OC-MCx	0.274	MCx-CI	0.660	MP-CI	0.697	RN-MP	0.918

ONLINE SUPPLEMENTARY FILE

Group 2

CD20/CD8		AT8/CD8		AT8/CD4		MG/CD8		MG/CD4		CD8/CD4	
Region	p	Region	p	Region	p	Region	p	Region	p	Region	p
MP-STN	0.930	MWM-MP	0.473	MWM-MP	0.473	STN-PUT	0.469	PUT-GP	0.456	PUT-MCx	0.964
MP-RN	0.638	MWM-STN	0.285	MWM-STN	0.285	STN-MP	0.208	PUT-STN	0.344	PUT-GP	0.793
MP-OC	0.490	MWM-GP	0.212	MWM-GP	0.212	STN-GP	0.207	PUT-SN	0.344	PUT-SN	0.688
MP-MWM	0.416	MWM-CI	0.169	MWM-CI	0.169	STN-TDM	0.154	PUT-TDM	0.064	PUT-CI	0.161
MP-TDM	0.230	MWM-SN	0.040	MWM-SN	0.040	STN-SN	0.153	PUT-MCx	0.059	PUT-MWM	0.142
MP-CI	0.094	MWM-TDM	0.031	MWM-TDM	0.031	STN-MWM	0.180	PUT-OC	0.023	PUT-TDM	0.090
MP-MCx	0.013	MWM-PUT	0.023	MWM-PUT	0.023	STN-CI	0.129	PUT-CI	0.018	PUT-OC	0.065
MP-SN	0.003	MWM-OC	0.019	MWM-OC	0.019	STN-OC	0.106	PUT-MWM	0.023	PUT-STN	0.056
MP-GP	0.001	MWM-RN	0.008	MWM-RN	0.008	STN-RN	0.074	PUT-RN	0.002	PUT-RN	0.029
MP-PUT	0.000	MWM-MCx	0.001	MWM-MCx	0.001	STN-MCx	0.007	PUT-MP	0.001	PUT-MP	0.014
STN-RN	0.702	MP-STN	0.695	MP-STN	0.695	PUT-MP	0.593	GP-STN	0.919	MCx-GP	0.843
STN-OC	0.545	MP-GP	0.516	MP-GP	0.516	PUT-GP	0.540	GP-SN	0.919	MCx-SN	0.754
STN-MWM	0.463	MP-CI	0.453	MP-CI	0.453	PUT-TDM	0.483	GP-TDM	0.361	MCx-CI	0.224
STN-TDM	0.266	MP-SN	0.134	MP-SN	0.134	PUT-SN	0.480	GP-MCx	0.296	MCx-MWM	0.194
STN-CI	0.112	MP-TDM	0.106	MP-TDM	0.106	PUT-MWM	0.488	GP-OC	0.189	MCx-TDM	0.141
STN-MCx	0.016	MP-PUT	0.083	MP-PUT	0.083	PUT-CI	0.414	GP-CI	0.159	MCx-OC	0.106
STN-SN	0.004	MP-OC	0.068	MP-OC	0.068	PUT-OC	0.361	GP-MWM	0.163	MCx-STN	0.096
STN-GP	0.002	MP-RN	0.030	MP-RN	0.030	PUT-RN	0.289	GP-RN	0.048	MCx-RN	0.056
STN-PUT	0.000	MP-MCx	0.006	MP-MCx	0.006	PUT-MCx	0.041	GP-MP	0.022	MCx-MP	0.032
RN-OC	0.815	STN-GP	0.765	STN-GP	0.765	MP-GP	0.893	STN-OC	0.177	GP-SN	0.923
RN-MWM	0.695	STN-CI	0.712	STN-CI	0.712	MP-TDM	0.868	STN-RN	0.036	GP-CI	0.315
RN-TDM	0.466	STN-SN	0.268	STN-SN	0.268	MP-SN	0.864	SN-CI	0.145	GP-MWM	0.271
RN-CI	0.224	STN-TDM	0.222	STN-TDM	0.222	MP-MWM	0.830	SN-MP	0.015	GP-TDM	0.209
RN-MCx	0.039	STN-PUT	0.179	STN-PUT	0.179	MP-CI	0.766	STN-TDM	0.363	GP-OC	0.159
RN-SN	0.013	STN-OC	0.149	STN-OC	0.149	MP-OC	0.694	STN-CI	0.145	GP-STN	0.147
RN-GP	0.006	STN-RN	0.076	STN-RN	0.076	MP-RN	0.599	SN-OC	0.177	GP-RN	0.090
RN-PUT	0.000	STN-MCx	0.016	STN-MCx	0.016	MP-MCx	0.118	SN-RN	0.036	GP-MP	0.054
OC-MWM	0.863	GP-CI	0.967	GP-CI	0.967	GP-TDM	0.988	STN-SN	1.000	SN-CI	0.312
OC-TDM	0.636	GP-SN	0.489	GP-SN	0.489	GP-SN	0.985	SN-MWM	0.153	SN-MWM	0.267
OC-CI	0.340	GP-TDM	0.427	GP-TDM	0.427	GP-MWM	0.941	STN-MCx	0.297	SN-TDM	0.195
OC-MCx	0.071	GP-PUT	0.367	GP-PUT	0.367	GP-CI	0.891	STN-MP	0.015	SN-OC	0.145
OC-SN	0.029	GP-OC	0.315	GP-OC	0.315	GP-OC	0.823	SN-TDM	0.363	SN-STN	0.130
OC-GP	0.013	GP-RN	0.197	GP-RN	0.197	GP-RN	0.737	SN-MCx	0.297	SN-RN	0.074
OC-PUT	0.000	GP-MCx	0.054	GP-MCx	0.054	GP-MCx	0.192	STN-MWM	0.153	SN-MP	0.041
MWM-TDM	0.795	CI-SN	0.481	CI-SN	0.481	TDM-SN	0.996	TDM-MCx	0.817	CI-MWM	0.862
MWM-CI	0.477	CI-TDM	0.414	CI-TDM	0.414	TDM-MWM	0.947	TDM-OC	0.639	CI-TDM	0.805
MWM-MCx	0.127	CI-PUT	0.351	CI-PUT	0.351	TDM-CI	0.891	TDM-CI	0.566	CI-OC	0.663
MWM-SN	0.067	CI-OC	0.297	CI-OC	0.297	TDM-OC	0.816	TDM-MWM	0.539	CI-STN	0.648
MWM-GP	0.030	CI-RN	0.176	CI-RN	0.176	TDM-RN	0.719	TDM-RN	0.234	CI-RN	0.471
MWM-PUT	0.001	CI-MCx	0.044	CI-MCx	0.044	TDM-MCx	0.157	TDM-MP	0.126	CI-MP	0.331
TDM-CI	0.611	SN-TDM	0.908	SN-TDM	0.908	SN-MWM	0.951	MCx-OC	0.844	MWM-TDM	0.961
TDM-MCx	0.158	SN-PUT	0.814	SN-PUT	0.814	SN-CI	0.895	MCx-CI	0.771	MWM-OC	0.818
TDM-SN	0.079	SN-OC	0.713	SN-OC	0.713	SN-OC	0.819	MCx-MWM	0.726	MWM-STN	0.808
TDM-GP	0.035	SN-RN	0.504	SN-RN	0.504	SN-RN	0.722	MCx-RN	0.405	MWM-RN	0.626
TDM-PUT	0.001	SN-MCx	0.156	SN-MCx	0.156	SN-MCx	0.158	MCx-MP	0.255	MWM-MP	0.473
CI-MCx	0.358	TDM-PUT	0.905	TDM-PUT	0.905	MWM-CI	0.953	OC-CI	0.918	TDM-OC	0.840
CI-SN	0.231	TDM-OC	0.798	TDM-OC	0.798	MWM-OC	0.885	OC-MWM	0.859	TDM-STN	0.828
CI-GP	0.109	TDM-RN	0.580	TDM-RN	0.580	MWM-RN	0.798	OC-RN	0.493	TDM-RN	0.625
CI-PUT	0.007	TDM-MCx	0.188	TDM-MCx	0.188	MWM-MCx	0.218	OC-MP	0.309	TDM-MP	0.455
MCx-SN	0.872	PUT-OC	0.889	PUT-OC	0.889	CI-OC	0.926	CI-MWM	0.934	OC-STN	0.993
MCx-GP	0.523	PUT-RN	0.665	PUT-RN	0.665	CI-RN	0.832	CI-RN	0.562	OC-RN	0.785
MCx-PUT	0.127	PUT-MCx	0.227	PUT-MCx	0.227	CI-MCx	0.208	CI-MP	0.362	OC-MP	0.601
SN-GP	0.590	OC-RN	0.779	OC-RN	0.779	OC-RN	0.907	MWM-RN	0.653	STN-RN	0.785
SN-PUT	0.127	OC-MCx	0.292	OC-MCx	0.292	OC-MCx	0.241	MWM-MP	0.450	STN-MP	0.596
GP-PUT	0.408	RN-MCx	0.412	RN-MCx	0.412	RN-MCx	0.274	RN-MP	0.733	RN-MP	0.796

ONLINE SUPPLEMENTARY FILE

Group 3											
CD20/CD8		AT8/CD8		AT8/CD4		MG/CD8		MG/CD4		CD8/CD4	
Region	p	Region	p	Region	p	Region	p	Region	p	Region	p
OC-CI	0.814	MP-MWM	0.755	SN-PUT	0.289	PUT-GP	0.832	SN-PUT	0.840	SN-PUT	0.382
OC-MWM	0.627	MP-RN	0.534	SN-MP	0.183	PUT-MWM	0.668	SN-GP	0.096	SN-STN	0.053
OC-RN	0.575	MP-GP	0.527	SN-RN	0.090	PUT-OC	0.530	SN-OC	0.035	SN-GP	0.036
OC-MP	0.314	MP-PUT	0.361	SN-MWM	0.116	PUT-RN	0.445	SN-RN	0.027	SN-TDM	0.017
OC-STN	0.265	MP-STN	0.248	SN-GP	0.075	PUT-STN	0.194	SN-MWM	0.039	SN-OC	0.015
OC-TDM	0.123	MP-CI	0.198	SN-STN	0.071	PUT-CI	0.192	SN-STN	0.016	SN-MWM	0.020
OC-MCx	0.078	MP-TDM	0.101	SN-TDM	0.024	PUT-MP	0.129	SN-TDM	0.007	SN-MP	0.010
OC-GP	0.052	MP-OC	0.090	SN-OC	0.013	PUT-MCx	0.080	SN-MP	0.002	SN-RN	0.009
OC-SN	0.002	MP-SN	0.076	SN-CI	0.017	PUT-TDM	0.032	SN-CI	0.003	SN-MCx	0.015
OC-PUT	0.002	MP-MCx	0.003	SN-MCx	0.000	PUT-SN	0.026	SN-MCx	0.001	SN-CI	0.014
CI-MWM	0.817	MWM-RN	0.798	PUT-MP	0.827	GP-MWM	0.821	PUT-GP	0.159	PUT-STN	0.307
CI-RN	0.782	MWM-GP	0.780	PUT-RN	0.569	GP-OC	0.683	PUT-OC	0.068	PUT-GP	0.240
CI-MP	0.494	MWM-PUT	0.591	PUT-MWM	0.585	GP-RN	0.586	PUT-RN	0.055	PUT-TDM	0.158
CI-STN	0.426	MWM-STN	0.447	PUT-GP	0.488	GP-STN	0.277	PUT-MWM	0.070	PUT-OC	0.143
CI-TDM	0.242	MWM-CI	0.366	PUT-STN	0.474	GP-CI	0.270	PUT-STN	0.034	PUT-MWM	0.149
CI-MCx	0.157	MWM-TDM	0.235	PUT-TDM	0.268	GP-MP	0.194	PUT-TDM	0.017	PUT-MP	0.110
CI-GP	0.120	MWM-OC	0.216	PUT-OC	0.188	GP-MCx	0.122	PUT-MP	0.005	PUT-RN	0.101
CI-SN	0.008	MWM-SN	0.190	PUT-CI	0.181	GP-TDM	0.054	PUT-CI	0.007	PUT-MCx	0.123
CI-PUT	0.008	MWM-MCx	0.016	PUT-MCx	0.008	GP-SN	0.045	PUT-MCx	0.003	PUT-CI	0.113
MWM-RN	0.979	RN-GP	0.972	MP-RN	0.715	MWM-OC	0.878	GP-OC	0.717	STN-GP	0.878
MWM-MP	0.665	RN-PUT	0.752	MP-MWM	0.721	MWM-RN	0.778	GP-RN	0.646	STN-TDM	0.726
MWM-STN	0.580	RN-STN	0.577	MP-GP	0.616	MWM-STN	0.418	GP-MWM	0.639	STN-OC	0.687
MWM-TDM	0.357	RN-CI	0.471	MP-STN	0.600	MWM-CI	0.401	GP-STN	0.479	STN-MWM	0.640
MWM-MCx	0.237	RN-TDM	0.308	MP-TDM	0.355	MWM-MP	0.318	GP-TDM	0.350	STN-MP	0.590
MWM-GP	0.189	RN-OC	0.283	MP-OC	0.253	MWM-MCx	0.206	GP-MP	0.179	STN-RN	0.563
MWM-SN	0.017	RN-SN	0.248	MP-CI	0.239	MWM-TDM	0.110	GP-CI	0.178	STN-MCx	0.570
MWM-PUT	0.016	RN-MCx	0.019	MP-MCx	0.012	MWM-SN	0.095	GP-MCx	0.112	STN-CI	0.542
RN-MP	0.656	GP-PUT	0.787	RN-MWM	0.981	OC-RN	0.887	OC-RN	0.919	GP-TDM	0.848
RN-STN	0.565	GP-STN	0.614	RN-GP	0.880	OC-STN	0.471	OC-MWM	0.888	GP-OC	0.808
RN-TDM	0.327	GP-CI	0.507	RN-STN	0.863	OC-CI	0.451	OC-STN	0.710	GP-MWM	0.748
RN-MCx	0.210	GP-TDM	0.345	RN-TDM	0.575	OC-MP	0.355	OC-TDM	0.552	GP-MP	0.704
RN-GP	0.161	GP-OC	0.319	RN-OC	0.437	OC-MCx	0.226	OC-MP	0.308	GP-RN	0.675
RN-SN	0.010	GP-SN	0.283	RN-CI	0.399	OC-TDM	0.114	OC-CI	0.295	GP-MCx	0.673
RN-PUT	0.010	GP-MCx	0.025	RN-MCx	0.029	OC-SN	0.096	OC-MCx	0.194	GP-CI	0.643
MP-STN	0.883	PUT-STN	0.815	MWM-GP	0.908	RN-STN	0.559	RN-MWM	0.962	TDM-OC	0.957
MP-TDM	0.593	PUT-CI	0.684	MWM-STN	0.891	RN-CI	0.532	RN-STN	0.783	TDM-MWM	0.880
MP-MCx	0.398	PUT-TDM	0.506	MWM-TDM	0.625	RN-MP	0.433	RN-TDM	0.622	TDM-MP	0.845
MP-GP	0.331	PUT-OC	0.474	MWM-OC	0.493	RN-MCx	0.279	RN-MP	0.358	TDM-RN	0.813
MP-SN	0.032	PUT-SN	0.427	MWM-CI	0.447	RN-TDM	0.150	RN-CI	0.340	TDM-MCx	0.799
MP-PUT	0.031	PUT-MCx	0.047	MWM-MCx	0.046	RN-SN	0.128	RN-MCx	0.228	TDM-CI	0.766
STN-TDM	0.714	STN-CI	0.854	GP-STN	0.983	STN-CI	0.947	MWM-STN	0.837	OC-MWM	0.919
STN-MCx	0.495	STN-TDM	0.673	GP-TDM	0.698	STN-MP	0.866	MWM-TDM	0.688	OC-MP	0.887
STN-GP	0.426	STN-OC	0.636	GP-OC	0.551	STN-MCx	0.610	MWM-MP	0.429	OC-RN	0.855
STN-SN	0.056	STN-SN	0.581	GP-CI	0.498	STN-TDM	0.425	MWM-CI	0.401	OC-MCx	0.838
STN-PUT	0.052	STN-MCx	0.078	GP-MCx	0.049	STN-SN	0.381	MWM-MCx	0.284	OC-CI	0.804
TDM-MCx	0.720	CI-TDM	0.833	STN-TDM	0.715	CI-MP	0.927	STN-TDM	0.842	MWM-MP	0.977
TDM-GP	0.646	CI-OC	0.795	STN-OC	0.566	CI-MCx	0.672	STN-MP	0.543	MWM-RN	0.948
TDM-SN	0.108	CI-SN	0.738	STN-CI	0.511	CI-TDM	0.490	STN-CI	0.502	MWM-MCx	0.924
TDM-PUT	0.099	CI-MCx	0.131	STN-MCx	0.051	CI-SN	0.445	STN-MCx	0.361	MWM-CI	0.892
MCx-GP	0.939	TDM-OC	0.957	TDM-OC	0.829	MP-MCx	0.714	TDM-MP	0.670	MP-RN	0.968
MCx-SN	0.267	TDM-SN	0.892	TDM-CI	0.740	MP-TDM	0.512	TDM-CI	0.614	MP-MCx	0.940
MCx-PUT	0.242	TDM-MCx	0.155	TDM-MCx	0.095	MP-SN	0.461	TDM-MCx	0.450	MP-CI	0.906
GP-SN	0.278	OC-SN	0.935	OC-CI	0.893	MCx-TDM	0.817	MP-CI	0.908	RN-MCx	0.969
GP-PUT	0.252	OC-MCx	0.170	OC-MCx	0.141	MCx-SN	0.760	MP-MCx	0.714	RN-CI	0.935
SN-PUT	0.917	SN-MCx	0.194	CI-MCx	0.215	TDM-SN	0.935	CI-MCx	0.817	MCx-CI	0.968

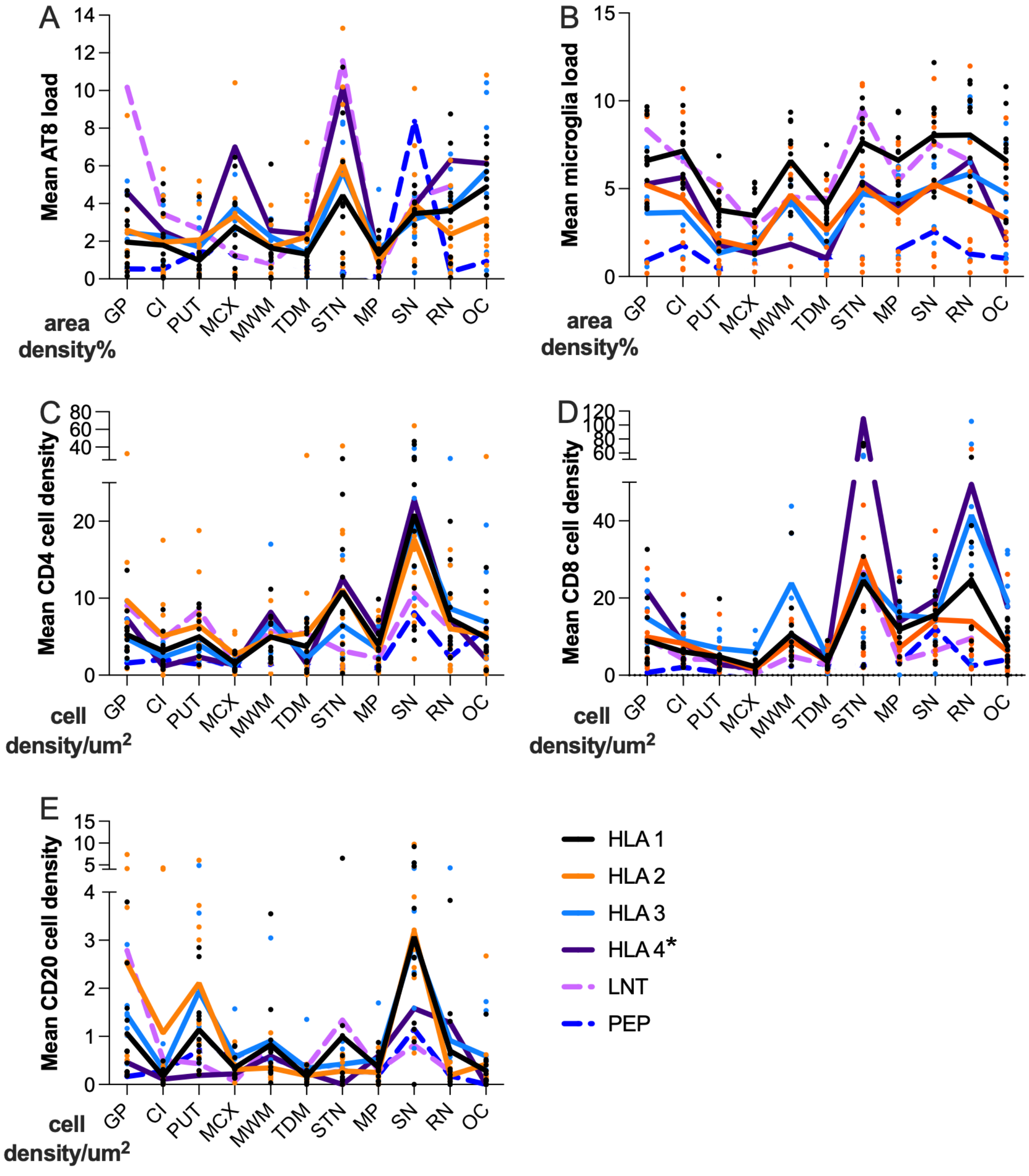

Figure S4 (legend: see next page)

Figure S4. Anatomical lesion profile of neuropathological variables.
The lesion profiles demonstrate differences in the patterns between all cases included in the study across HLA groups 1–3 (see manuscript). The LNT case (removed from Group 2; note that OC was not available) and the suspected PEP case (removed from Group 3; note that MCX, MWM, TDM and STN was not available) are shown separately, and the single IgLON5-HLA haplotype (*DRB1**10:01-*DQB1**05:01) associated case is designated as HLA group 4*. Dots represent individual case values within each group, and lines indicate group means or for single cases connecting regional values. **A**. Mean AT8 (p-tau) load (area density %), **B.** mean microglial load (area density %), **C.** mean CD4 cell density/mm2, **D.** mean CD8 cell density/mm2 and **E.** mean CD20 cell density/mm2 across HLA groups, LNT and PEP cases, and brain regions. Abbreviations: GP, globus pallidus; CI, capsula interna; PUT, putamen; MCX, motor cortex; MWM, motor white matter; TDM, thalamus dorsomedial nucleus; STN, subthalamic nucleus; RN, red nucleus; MP, midbrain peduncles; SN, substantia nigra; OC, oculomotor complex. N for each group is as follows: HLA 1: 12; HLA 2: 10; HLA 3: 7, HLA 4*, LNT and PEP: 1.

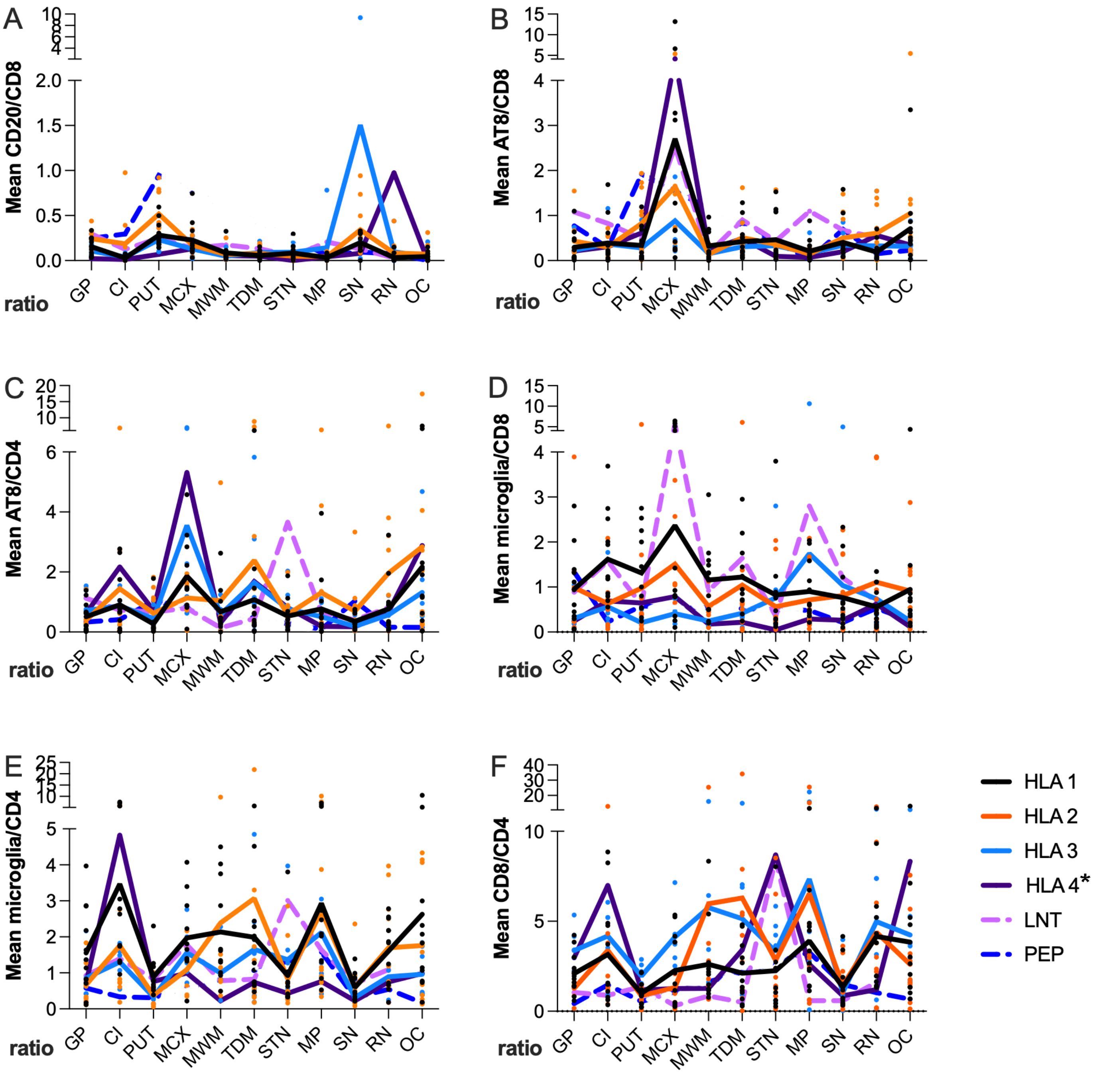

Figure S5 (legend: see next page)

Figure S5. Anatomical lesion profile of ratios of neuropathological variables.
The lesion profiles demonstrate differences in the patterns between all cases included in the study across HLA groups 1–3 (see manuscript). The LNT case (removed from Group 2; note that OC was not available) and the suspected PEP case (removed from Group 3; note that MCX, MWM, TDM and STN was not available) are shown separately, and the single IgLON5-HLA haplotype (*DRB1**10:01-*DQB1**05:01) associated case is designated as HLA group 4*. Dots represent individual case values within each group, and lines indicate group means or for single cases connecting regional values. **A**. Mean ratio of CD20/CD8 cell density/mm2, **B.** mean ratio of AT8 area density/CD8 cell density/mm2, **C.** mean ratio of AT8 area density/CD4 cell density/mm2, **D.** mean ratio of microglia area density/CD8 cell density/mm2, **E.** mean ratio of microglia area density/CD4 cell density/mm2, and **F.** mean ratio of CD8/CD4 cell density/mm2 across HLA groups, LNT and PEP cases, and brain regions. The ratio of AT8 and microglia area density to CD4 and CD8 cell density (per μm^2) reflects the average tau or microglial burden per CD4 or CD8 lymphocyte. This does not imply that lymphocytes express p-tau or microglial markers; rather, it indicates whether tau or microglial pathology is associated with a higher or lower number of T cells per unit of pathology within each anatomical region across HLA haplotype groups. Abbreviations: GP, globus pallidus; CI, capsula interna; PUT, putamen; MCX, motor cortex; MWM, motor white matter; TDM, thalamus dorsomedial nucleus; STN, subthalamic nucleus; RN, red nucleus; MP, midbrain peduncles; SN, substantia nigra; OC, oculomotor complex. N for each group is as follows: HLA 1: 12; HLA 2: 10; HLA 3: 7, HLA 4*, LNT and PEP: 1.
